# Metabolomics of *Apc*^*Min/+*^ mice genetically susceptible to intestinal cancer

**DOI:** 10.1186/1752-0509-8-72

**Published:** 2014-06-23

**Authors:** Jean-Eudes J Dazard, Yana Sandlers, Stephanie K Doerner, Nathan A Berger, Henri Brunengraber

**Affiliations:** 1 Center for Proteomics and Bioinformatics, Case Western Reserve University School of Medicine, Cleveland, OH 44106, USA; 2 Department of Nutrition, Case Western Reserve University School of Medicine, Cleveland, OH 44106, USA; 3 Kennedy Krieger Institute, Baltimore, MA 21205, USA; 4 Department of Genetics, Case Western Reserve University School of Medicine, Cleveland, OH 44106, USA; 5 Department of Medicine, Case Western Reserve University School of Medicine, Cleveland, OH 44106, USA; 6 Case Comprehensive Cancer Center, Case Western Reserve University School of Medicine, Cleveland, Ohio 44106, USA

**Keywords:** Metabolomics, Fat diet, Tumor development, Association and correlation analysis, High-throughput mass spectrometry

## Abstract

**Background:**

To determine how diets high in saturated fat could increase polyp formation in the mouse model of intestinal neoplasia, *Apc*^*Min/+*^, we conducted large-scale metabolome analysis and association study of colon and small intestine polyp formation from plasma and liver samples of *Apc*^*Min/+*^ vs. wild-type littermates, kept on low vs. high-fat diet. Label-free mass spectrometry was used to quantify untargeted plasma and acyl-CoA liver compounds, respectively. Differences in contrasts of interest were analyzed statistically by unsupervised and supervised modeling approaches, namely Principal Component Analysis and Linear Model of analysis of variance. Correlation between plasma metabolite concentrations and polyp numbers was analyzed with a zero-inflated Generalized Linear Model.

**Results:**

Plasma metabolome in parallel to promotion of tumor development comprises a clearly distinct profile in *Apc*^*Min/+*^ mice vs. wild type littermates, which is further altered by high-fat diet. Further, functional metabolomics pathway and network analyses in *Apc*^*Min/+*^ mice on high-fat diet revealed associations between polyp formation and plasma metabolic compounds including those involved in amino-acids metabolism as well as nicotinamide and hippuric acid metabolic pathways. Finally, we also show changes in liver acyl-CoA profiles, which may result from a combination of *Apc*^*Min/+*^-mediated tumor progression and high fat diet. The biological significance of these findings is discussed in the context of intestinal cancer progression.

**Conclusions:**

These studies show that high-throughput metabolomics combined with appropriate statistical modeling and large scale functional approaches can be used to monitor and infer changes and interactions in the metabolome and genome of the host under controlled experimental conditions. Further these studies demonstrate the impact of diet on metabolic pathways and its relation to intestinal cancer progression. Based on our results, metabolic signatures and metabolic pathways of polyposis and intestinal carcinoma have been identified, which may serve as useful targets for the development of therapeutic interventions.

## Background

In the high-throughput omics era, metabolomics is a rapidly emerging field that involves non-targeted, comprehensive analysis of known and unknown small biomolecules in a given biological sample [1-4]. While the metabolome is strongly influenced by multiple factors including heredity, diet, disease progression and response to therapy, this metabolomics approach also allows for global assessment of biological variations as a consequence of such variation in genetics or environment. Changes in the metabolome can be used to elucidate changes that occur downstream of genomic or proteomic pathways. These changes can further be correlated with alterations or interventions associated with particular biochemical pathways, disease stage or environmental factors [5,6]. Metabolomics is a snapshot of the metabolic status of a living system at a specific biological time point. Unlike changes in the genome or proteome, metabolic fluctuations take place in a shorter time frame, therefore metabolomics may assist in early diagnosis or real time monitoring of disease [7].

Metabolomics promises to be a powerful systems approach for studying metabolic profiles pertinent to a variety of normal and disease states. Global metabolic profiling is widely used in clinical studies to assist in early diagnosis or real time monitoring of disease [7], for identifying biomarkers for neuropsychiatric [8], cardiovascular [9,10] and liver diseases [11], colorectal neoplasia [12], and for characterization of dysregulation in some metabolic pathways [13,14]. Metabolomics based methods have also been applied in translational studies to characterize and understand genetically modified rodent models of different disease [15,16]. Application of metabolomics has also been shown to be advantageous in drug development, discovery and toxicology fields [17,18]. Metabolomics based methods have also been applied in translational studies to characterize and understand genetically modified rodent models of different disease [15,16]. Although all these studies are data driven rather than hypothesis driven, metabolomics widen the fields in which hypothesis can be formulated and increase the potential to uncover unexpected correlations and new insights into biological processes.

Cancer progression and development affects the whole metabolome. Metabolomics of cancer tissues can give insight into mechanisms surrounding carcinogenesis and can help identify cancer biomarkers for establishing preventive and therapeutic treatments [19-21]. Recently, several cancer metabolomics studies were carried out in a variety of cancers and used as a diagnostic or disease pattern recognition tool and as an assessment tool for different anti-cancer therapies [22-27]. This approach has great potential in clinical studies [28] such as for tumor typing and biomarker discovery [29].

Prevention of colon cancer remains a significant public health issue that is highly associated with genetic and environmental factors such as diet composition [30]. Evidence is emerging that diet and nutrient factors may play an important role in colorectal cancer incidence and progression [31-34]. Consumption of high fat diet in combination with genetic factors leads to energy imbalances and increased risk of colon cancer [35-39]. Meanwhile, it was also demonstrated that obesity and excess body weight is a major risk factor for colon cancer [40,41]. Several hypotheses have emerged to explain the positive correlation between increased adiposity and colorectal cancer. Some recent studies reported that obesity induced insulin resistance and chronic inflammation lead to hyperglycemia and hyperlipemia [42]. These have been positively associated with colon cancer risk and development [40,43].

Previous studies have investigated whether a high fat diet promotes formation and development of intestinal polyps in mice genetically predisposed to colon cancer. Specifically, studies have investigated the interaction of fat content in the diet and genetic susceptibility to colon cancer in the *Apc*^*Min/+*^ mouse model. Because mutations in Adenomatous Polyposis Coli (APC) was observed in over 80% of sporadic human colon cancer cases [44-46], *Apc*^*Min/+*^ mice carrying a dominant mutation in the *Apc* gene commonly serve as the mouse model of choice for the human Familial Adenomatous Polyposis (FAP) syndrome. *Apc*^*Min/+*^ mice spontaneously develop multiple intestinal neoplasia (*Min*) and numerous intestinal polyps, which increase in number and accelerate in development in response to a high fat diet [47]. Also, we recently showed that high-fat dietary exposure can increase intestinal polyp formation in the *Apc*^*Min/+*^ model by several fold (>5) as well as both systemic and local inflammation before the onset of overt obesity or characteristics associated with metabolic syndrome, such as increase insulin or glucose levels [48].

The first phase of this study was to run untargeted metabolomics profile and association analyses to a clinical outcome on plasma samples (assayed by GC-MS) from wild type and *Apc*^*Min/+*^ mice fed either with high fat or low fat diets. Acyl-CoA profiles (assayed by LC-MS/MS) on liver tissue samples were analyzed for all groups as well. The plasma metabolic concentrations found to be statistically different between mutation and diet factors, and statistically correlated to intestinal polyp number, was then studied by large scale functional metabolomics approaches. Metabolomic pathway and network analyses reveal an association in the presence of high fat diet and *Apc* mutation between polyp formation and plasma concentrations of metabolic compounds including those involved in the metabolic pathways of several amino-acids, hippuric acid, and nicotinamide. Liver acyl-CoA profiles also show changes, which may result from a combination of *Apc*^*Min/+*^-mediated cancer progression and high fat diet. These results illustrate that high-throughput mass spectrometry-based metabolomics combined with appropriate statistical modeling and large scale functional metabolomics approaches can be used to investigate complex environment-gene interactions, such as a combined diet-mutation effect, and their association with intestinal polyposis and tumorigenesis. Understanding these important interactions in biological systems can potentially lead to the identification of new biomarkers or the development of early diagnostic tools.

## Methods

### Animal experimental design

#### Ethics statement

This study was carried out in strict accordance with the recommendations in the Guide for the Care and Use of Laboratory Animals of the National Institutes of Health. Procedures were approved and conducted in compliance with Institutional Animal Care and Use Committee (IACUC) standards at Case Western Reserve University (IACUC, Protocol 2012–0080). All surgery was performed under 2% isoflurane anesthesia, and all efforts were made to minimize suffering.

#### Mice strains

Wild-type C57BL/6 J (B6 *Apc*^*+/+*^) and mutant C57BL/6 J-*Apc*^*Min/+*^/J (B6 *Apc*^*Min/+*^) mice were purchased from The Jackson Laboratory (Bar Harbor, ME) and maintained on a 12-h light/dark cycle at the Wolstein Research Facility (CWRU).

#### Diet composition and tissue sample harvesting

The animal experimental design was as previously described in the literature in the field using the same animal model [48,49]. High- or low-saturated fat diets were as previously described [31,48]. Briefly, diets were purchased from Research Diets and contained identical amounts of vitamins, minerals and protein. The high fat (58%) and low fat (10.5%) diets were made using hydrogenated coconut oil for the fat source. Male mice were maintained on normal laboratory diet from birth till 30 days of age, then randomly placed on a high or low fat diet and maintained on this diet for 60 days until dissection and tissue harvesting. At this time (90 days of age), samples were collected from all mice; polyp formation (or non-formation) was assessed by direct observation and confirmed by histology. When present at this time, polyps were counted and measured in size and mass per animal.

#### Sample preparations

Powdered frozen liver tissue (25 mg - spiked with internal standards (5 nmol) of heptadecanoic acid and [^2^H_27_]myristic acid as a retention time locker compound) was extracted with 2 ml of CH_3_CN/Methanol (1:1 precooled at −12°C and degassed with N_2_ flow) using Polytron homogenizer. The slurry was centrifuged at 3800 rpm, 4°C. The supernatant was collected, dried with air before derivatization. 25 μl of plasma was spiked with 5 nmol heptadecanoic acid. Metabolites were extracted with 0.5 ml of acetonitrile/methanol (1:1 precooled at −12°C and degassed with N_2_). Samples were centrifuged at 3800 rpm, 4°C. The supernatant was collected, dried with air before derivatization. 30 μl of 15 mg/ml of methoxylamine-HCl in dry pyridine was added to samples and incubated at 30°C for 90 minutes, followed by 70 μl of N-Methyl-N-trimethylsilyltrifluoroacetamide with 1% trimethylchlorosilane. The mixture was incubated at 37°C for 40 min.

### GC-MS analyses

#### Mass spectrometry

All solvents, standards and labeled internal standards and derivatization reagents for GC-MS were obtained from Sigma-Aldrich. GC-MS analyses were carried out on an Agilent 5973 mass spectrometer, linked to a model 6890 gas chromatograph equipped with an autosampler, a Phenomenex ZB-5MSi capillary column (30 m, 0.25 mm inner diameter, 0.25 μm film thickness). The carrier gas was helium (1.67 psi) and injections were 1 μl in splitless mode. The GC temperature program was: initial temperature 60°C, hold for 1 min, increase by 10°C/min to 325°C and hold 10 min. The injector temperature was set at 250°C and the transfer line at 275°C. EI source and quadrupole temperatures were set at 250°C and 150°C, respectively.

#### GC-MS metabolite identification and quantification

Peak extraction from raw GC/MS data was carried out according to Stein’s initial method [50] implemented in the Automated Mass spectral Deconvolution and Identification System (AMDIS) software developed at the National Institute of Standards and Technology (NIST, http://chemdata.nist.gov/dokuwiki/doku.php?id=chemdata:amdis). Peak identification was carried out by matching retention time and mass spectral similarity against homemade and Fiehn libraries (Agilent). For quantification of peak areas, the data was exported to the SpectConnect server developed by Styczynski *et al*. [51] at Massachusetts Institute of Technology (MIT, http://spectconnect.mit.edu/). From the output of SpectConnect, the only metabolites retained were those that were consistently detected in at least 80% of samples. All peak areas were normalized relative to the peak area of the internal standard heptadecanoic acid. All relative amounts then were normalized to the relative concentration of the corresponding metabolites in the control sample.

### LC-MS analyses

#### Sample preparation

Acyl-CoA profiles were assayed by LC-MS analyses. Samples of 200–300 mg of frozen and powdered liver tissues were spiked with an internal standard of [^2^H_9_]pentanoyl-CoA and were extracted with methanol-H_2_O, 5% v/v acetic acid buffer (5 ml) using a Polytron homogenizer. The slurry was centrifuged at 4000 rpm at 4°C. The supernatant was collected and loaded on a SPE cartridge (SupelCo 3 ml cartriges 2-(2-pyridyl) ethyl functionalized silica gel). Cartridges were washed with 9 ml of methanol-H_2_O, 5% v/v acetic acid buffer, Acyl-CoAs were eluted with 9 ml of 50 mM ammonium formate in MeOH-H_2_O (1:1), followed by 9 ml of 50 mM ammonium formate in methanol-H_2_O (3:1) and 9 ml of methanol. The slurry was dried with air and the residue dissolved in 100 μL of buffer A.

#### HPLC separation

For short and medium chain acyl-CoAs HPLC separation we used a Thermo Hypersil Gold column (2 mm × 100 mm 3 μm) and a 2 × 4 mm guard column with the same packing material. The flow rate was 200 μl/min in gradient mode with the following method: the column was equilibrated with 98% mobile phase A (CH_3_CN/H_2_O 98:2 v/v, containing 50 mM ammonium formate). After sample injection, 98% mobile phase A was continued for 3 min, followed by a 23 min gradient to 90% of buffer B (H_2_O/CH_3_CN 98:2 v/v, containing 50 mM ammonium formate). The column was held at 90% B for 5 min, followed by wash by a 10 min. gradient to starting conditions (98% buffer A). For long chain acyl-CoAs, the same column and flow rate were used in gradient mode with the following method: the column was equilibrated with 60% mobile phase A (CH_3_CN/H_2_O (98:2 v/v, containing 50 mM ammonium formate). After sample injection, the 60% mobile phase A was continued for 3 min, followed by a 28 min gradient to 90% of buffer B (H_2_O/CH_3_CN 98:2 v/v, containing 50 mM ammonium formate). The column was held at 90% B for 5 min, followed by a wash by a 10 min. gradient to starting conditions (60% buffer A - 40% buffer B).

#### Mass spectrometry

The analysis was performed with Applied Biosystems API 4000 QTrap (AB SCIEX). Nitrogen was used as nebulizer and desolvation gas. Declustering potential was 90 V and collision energy was set to 50 eV. Acyl CoAs were detected in multiple reaction monitoring (MRM) mode. Specific MRM transitions for all Acyl CoAs are provided in (Additional file 1: Table S1).

### Label-free data preprocessing

#### Raw data acquisition and quantitative processing

All raw GC-MS spectra were processed with AMDIS software and compared against Fiehn GC-MS library (Agilent). Extracted data were exported to the external server while peaks that were not consistently found in 80% of samples have been excluded from the data analysis. Through all the samples, 220 unique metabolites were detected. Out of these detected metabolites, 82 in total were fully annotated.

#### Data quality control and pre-filtering

After raw data acquisition and processing, data QC and pre-filtering were performed for this study. To reduce the number of variables (metabolites) at play, that is, to reduce the dimensionality of the data and error rates in subsequent inferences that are due to the lower number of samples than variables (*p* >> n paradigm), and to simultaneously remove the variables (metabolites) with the largest number of missing values without potentially inducing a severe selection bias in the presence of informative missingness (see next paragraph), an empirical variable selection procedure was carried out. Those metabolites were retained for which the observed count of *missing values per metabolite* is the nearest upper integer (ν) satisfying two criteria simultaneously: (i) ν maximizes the difference between the overall number of remaining metabolites after selection and the overall number of missing values, (ii) ν is less than the total sample size *n* minus the minimal half sample size (*n*_*g*_ / 2) over all experimental groups *g* = 1,…,*G* (see also example in reference [52]). Here, with an initial number of *p* = 220 metabolites, the procedure retained a final number of *p* = 201 metabolites. Out of these selected metabolites, 76 in total were fully annotated.

#### Missing value imputation

Missing values in LC/MS data arise because of imperfect detection and alignment of peak intensities or by true absence. To account for the non-random nature of the *missingness mechanism* at play and its extent in this type of data (informative missingness or non-ignorable left-censoring), we used a probability model adapted from Wang *et al.*[53] which describes ‘*artifactual missing events*’. This model makes inferences on the missing values of one sample based on the information from other ‘similar’ samples (technical replicates or nearest neighbors). It substitutes a missing measurement of intensity with its expected value of the true intensity given that it is unobservable. Remaining missing values represent truly absent metabolites in the samples and are typically imputed by taking an estimate of the background noise (see also reference [52]).

#### Transformation of features

To help remove sources of systematic variation in the measured intensities (bias and variance due to experimental artifacts) and to ensure that the usual assumption of normality is met for statistical inferences, we first applied a log-transformation on the variables (metabolites). In addition, since the homoscedasticity assumption in multi-group designs is also required, e.g. in ANOVA models, we also applied our recently developed ‘*joint adaptive mean-variance regularization*’ procedure as described in [54] now available as an R package called “MVR” [55]. Briefly, the joint adaptive regularization procedure simultaneously overcomes the lack of degrees of freedom and the variance-mean dependence issue in this type of dataset where the number of variables hugely dominates the number of sample [54]. The procedure stabilizes the variance and normalizes the concentration values, both of which are required for preprocessing high-dimensional data and making inferences. Although, the procedure is designed to stabilize the variance across variables (metabolites), we observed in our methodology article [54] that it also translates into good variance stabilization effect across sample groups in a multi-group design, as is the case in this study. Note that intensity levels on this transformed scale are on the entire real domain, so they are not necessary positive.

### Experimental design

#### Experimental units, groups, factors and sample size

In this experimental design three factors are at play: (i) the *Diet* (High Fat (HF) vs. Low Fat (LF)), denoted DF, (ii) the *Genotype* (*Apc* Wild-Type (WT) vs. Mutant (MU)), denoted GF, and (iii) the *Source of Tissue* (Plasma (PLA) vs. Liver (LIV)), denoted TF, representing the variable over which repeated measures are made within each experimental unit. The experimental units under study are the *n* = 20 individual mice or biological replicate. Samples were assumed to be *independent* and *randomly* sampled from the entire population. Further, samples were randomized across the design (without blocking) and balanced for each combination of *Diet* by *Genotype* by *Source of Tissue* experimental group (8) to have an equal number of biological replicates per experimental group (*n*_*g*_ = 5). The group sample sizes used in this study were consistent with other metabolomics studies carried out in the same mouse model (*n*_*g*_ = 2–10 [56]; *n*_*g*_ = 6–9 [49]). A common reference sample was used to normalize mass spectrometry readouts. No technical replicates were performed. No sample pooling was done. Observations were repeated with the same biological replicate for each tissue. In sum, this is a factorial arrangement of treatments (*Diet* by *Genotype*) laid out on a balanced Completely Randomized Design (CRD) with repeated measures on another treatment (*Source of Tissue*) amounting to a total of 2*n* = 40 observations (Table 1).

**Table 1:** Experimental design

		**Diet factor (DF)**			
		**Low fat (LF)**		**High fat (HF)**	
**Genotype Factor (GF)**	Wild-Type (WT)	**Source of Tissue Factor (TF)**		**Source of Tissue Factor (TF)**	
		Liver (LIV)	Plasma (PLA)	Liver (LIV)	Plasma (PLA)
		Mice #1-5	Mice #1-5	Mice #6-10	Mice #6-10
	*Apc* Mutant (MU)	**Source of Tissue Factor (TF)**		**Source of Tissue Factor (TF)**	
		Liver (LIV)	Plasma (PLA)	Liver (LIV)	Plasma (PLA)
		Mice #11-15	Mice #11-15	Mice #16-20	Mice #16-20

In all experimental groups *n*_*g*_ = 5.

### Statistical analyses

#### Analysis of variance of Acyl-CoA concentrations profiles of liver samples

The standard error of the means were computed for all absolute or relative concentration means per experimental group (*n*_*g*_ = 5). Two-sample two-sided t-test *p*-values, or multi-group ANOVA *p*-values, were computed for assessing the significance of difference between two groups, or between a group mean and the overall mean across the groups under the assumption of normality and homoscedasticity of concentration levels within each group.

#### Test of independence/association

We carried out a Pearson’s Chi-square test of independence to assess the independence between the two categorical factors (*Diet* by *Genotype*) of the experimental design. Basically, we tested the distribution of counts in a 2-by-2 contingency table to determine whether there were nonrandom associations between two categorical *factors*.

#### Principal Component Analysis of plasma samples

Potential groups and outliers among the samples were checked by a Principal Component Analysis (PCA) [57]. A PCA scatterplot of samples (scoreplot) was formed by plotting the plasma samples in the first two coordinate axes (PC1 and PC2) of the PC space. The scoreplot represents the scores that each sample has on the PCs. Points that have similar scores in the PC space cluster together and correspond to samples behaving similarly.

#### Statistical modeling and inference of differential metabolite concentrations in plasma samples

A standard analysis method in modeling high dimensional data is to fit the same statistical model individually to each variable (metabolite) and test for the contrast or effect of interest using the hypothesis testing framework. A drawback of this univariate approach is that the correlation structure or dependence between the variables is ignored. However, thanks to the parallel nature of the high-throughput data, some compensating possibilities exist by borrowing information across variables, resulting in more stable variance estimates, which in turn assist in inference about each variable individually. Statistical modeling was performed using a linear model of analysis of variance (mixed two-way ANOVA), fitted univariately to each individual variable (single metabolite) for the plasma samples. If we let *Y*_*ij*_ be the intensity signal on the transformed scale of the *j*^th^ variable (metabolite) and *i*^th^ unit (mice) using the appropriate transformation mentioned above [54], a linear ANOVA model for *each* individual metabolite *j* is fitted as follows:

$$Y_{\mathrm{ij}}=\mu_{j}+x_{\mathrm{ij}}^{T}\beta_{j}+\epsilon_{\mathrm{ij}}$$

where (for *each* individual metabolite *j*) *μ*_*j*_ represents the average signal intensity for that metabolite across *all* factors and observations; the vector of regresssors **x**_*ij*_ = [*G*_*ij*_, *D*_*ij*_, *G*_*ij*_ ⋅ *D*_*ij*_]^*T*^ represents the covariates (i.e. the factors of interest, all taken as fixed effects) with the *Genotype* factor denoted as *G*_*ij*_, the *Diet* factor as *D*_*ij*_ and their two-way interaction as *G*_*ij*_ ⋅ *D*_*ij*_; **β**_*j*_ = [*β*_1*j*_, *β*_2*j*_, *β*_3*j*_]^*T*^ is the vector of regression coefficients to be estimated; and the error term *ϵ*_*ij*_ represents the random deviations due to non-systematic sources of variations, assumed to be normally distributed with mean 0 and some (unknown) variance component: $\epsilon_{\mathrm{ij}}∼N\;(0,\sigma_{j}^{2})$. In ANOVA notation, the model can be written as *y*_*ijkl*_ = *μ*_*j*_ + (*G*)_*jk*_ + (*D*)_*jl*_ + (*GD*)_*jkl*_ + *ϵ*_*ijkl*_, where (*G*)_*jk*_, (*D*)_*jl*_, and (*GD*)_*jkl*_ represent the *Genotype* factor, *Diet* factor and their interaction, respectively. A number of authors have noted in gene expression studies that application of empirical Bayes methods and estimators derived from them (moderated *F*-, *t*-, and *B* statistics) are more reliable and resulted in greater statistical power [58-62]. In addition, posterior odds statistics have proven to be a useful means of ranking variables in terms of evidence for differential expression [59,62-65]. Information is borrowed by constraining the within-block correlations to be equal between variables and by using empirical Bayes methods to moderate the standard deviations between them [48,66]. These methods are particularly appropriate when only few samples are available, as is always the case in high throughput datasets [62].

#### Reports for label-free analysis in plasma samples

In this experimental design, contrasts were built for each of the fixed effects of interest, and coefficients were estimated accordingly. Variables were ranked in order of evidence of differential concentration. Corresponding *p*-values were adjusted for multiple testing using a recent extension of the standard Benjamini-Hochberg procedure, which controls the expected False Discovery Rate (FDR) [67]. This error rate, called the *positive* FDR (denoted *pFDR*) [68,69], results in a procedure that is less conservative than the FDR. (Additional file 1: Table S1; Additional file 2: Table S2 and Additional file 3: Table S3) report top-ranked metabolites (rows) from the model fit for each effect of interest. Each table consists in columns with the following information: the estimated log_2_-Fold Change (FC) or *M* log-ratio for individual metabolite across effect or contrast of interest. It represents a log_2_-FC (*M =* log_2_ (*FC*)) between two experimental conditions in the case of a main effect and to a difference in log_2_-FC in the case of an interaction effect. An estimated average log_2_-fold change of - 1 and + 1 correspond to a ½- and 2-fold change respectively. Moderated *t*- and *B*- statistics represent different measures of statistical significance. The Moderated *t*-statistic corresponds to the usual *t*-statistic except that information has been borrowed across variables (metabolites), while the *B*-statistic is the empirical Bayes log_2_ of the posterior odds that the metabolite is differentially expressed. Finally raw and adjusted *p*-values are listed. Note that in every list all the metabolites are ranked by adjusted *p*-value and then by *B-*statistic.

#### Modeling polyp counts

To analyze how experimental factors (*Diet* and *Genotype*) control the relationship between polyp counts and individual plasma metabolite concentrations, we modeled the polyp counts univariately for each variable (metabolite) using a zero-inflated negative-binomial regression model. This provides a way to account for the excess zeros in addition to allowing for overdispersion simultaneously [70,71]. Zero-inflated models are preferable to their classical Generalized Linear Model (GLM) counterparts (Poisson or Negative Binomial regression models) [72,73] to model these two situations typically occurring in biomedical sciences count data. Briefly, zero-inflated models are two-component mixture models combining (i) a zero-inflated count probability distribution (Binomial probability mass at 0), employed for zero counts, with (ii) a non-zero count probability distribution (e.g. Negative Binomial), employed for positive counts. Zero-inflated models allow distinct regressors for each component model. Formally, if we denote by *C*_*ij*_ the observed polyps count for the *i*^th^ unit (mice) and *j*^th^ variable (metabolite), the probability distribution of *C*_*ij*_ counts for *each* individual metabolite *j* can be written as:

$$\mathrm{Pr}\left(C_{\mathrm{ij}}=c_{\mathrm{ij}}|x_{\mathrm{ij}},z_{\mathrm{ij}}\right)=\pi_{\mathrm{ij}}I_{\left\{0\right\}}\left(c_{\mathrm{ij}}\right)+\left(1-\pi_{\mathrm{ij}}\right)f\left(c_{\mathrm{ij}}|x_{\mathrm{ij}}\right)$$

Where *I*_{0}_ (.) denotes the indicator function at 0, $\pi_{\mathrm{ij}}=\pi \left(z_{\mathrm{ij}}^{T}\gamma_{j}\right)$ denotes the unobserved probability of belonging to the zero-inflated count component, modelled by a binomial GLM model $\pi \left(z_{\mathrm{ij}}^{T}\gamma_{j}\right)=\exp\left(z_{\mathrm{ij}}^{T}\gamma_{j}\right)$ using the canonical log link function, and where the vectors of regressors **x**_*ij*_ and **z**_*ij*_, with corresponding coefficients **β**_*j*_ and **γ**_*j*_, are the vectors of covariates in the non-zero and zero-inflated count components, respectively. The corresponding regression equation for the mean count is $E\left[c_{\mathrm{ij}}|x_{\mathrm{ij}}\right]=\pi_{\mathrm{ij}}\cdot 0+\left(1-\pi_{\mathrm{ij}}\right)\exp\left(x_{\mathrm{ij}}^{T}\beta_{j}\right)$. Here, using above notations, we fit the zero-inflated model with covariates **x**_*ij*_ = [*G*_*ij*_, *D*_*ij*_, *G*_*ij*_ ⋅ *D*_*ij*_ ⋅ *Y*_*ij*_]^*T*^ (i.e. the factors of interest) and **z**_*ij*_ = 1 (i.e. intercept only for simplicity), where *G*_*ij*_ ⋅ *D*_*ij*_ ⋅ *Y*_*ij*_ denotes the three-way interaction between the *Genotype* factor *G*_*ij*_, the *Diet* factor *D*_*ij*_ and *Y*_*ij*_ the intensity signal on the transformed scale of the *j*^th^ metabolite and *i*^th^ mice.

#### Implementations, algorithms and softwares

Whenever available, implementations and algorithms of our methods are freely available from the CRAN consortium (Comprehensive R Archive Network) at http://cran.r-project.org/. All other R codes written in our group can be provided upon request. For linear modeling and supervised inferences, we used the package “limma” [62]. For count data modeling, we used the package “pscl” [48,74]. Finally, for the control of the positive FDR, we used the package “qvalue” [65].

### Functional metabolomics analyses

Significantly altered metabolites with *pFDR*-adjusted *p*-values ≤ 0.05 outputted from the statistical model were selected for functional metabolomics analyses. Metabolite identifiers with their corresponding raw and *pFDR*-adjusted *p*-values were up-loaded onto the Ingenuity Pathway Analysis application for biological functions, canonical metabolomics pathways, and interaction network analyses (IPA version #14855783, http://www.ingenuity.com/ - Ingenuity Systems, Inc., Mountain View, CA). Whenever a multiple-testing correction was required to assess significance e.g. of function, pathway, or network enrichment, we report adjusted *p*-values using the Benjamini-Hochberg (BH) method [67], which is the only error-rate control-procedure available for that matter in IPA at this time.

#### Canonical metabolomics pathways

For the computation of enrichment *p*-values, we used the IPA Metabolomics Knowledge Base as our reference database set, i.e. the universe of all metabolomics entities. Significance of each individual pathway was measured in two ways: (i) A ratio (in percentage) of the number of selected molecules mapping a selected pathway that meets a cutoff criterion, divided by the total number of molecules that exist in this canonical pathway. (ii) A right-tailed Fisher exact test *p*-value for the probability under the null hypothesis that the association between those metabolites found in a given pathway of our list with all those constitutive of the corresponding canonical metabolomics pathway is explained by chance alone (the null hypothesis being that the function/data set association is just random). The smaller the *p*-value, the less likely it is that the association is random and the more significant the association. Note that the right-tailed Fisher’s exact test only assesses over-represented pathways, that is, those that have more molecules found than would be expected by chance alone.

#### Metabolomics network analysis

Significant metabolites were mapped in IPA to the global molecular network that was developed from the Ingenuity Knowledge Base. Networks for these metabolites were algorithmically generated based on their connectivity. A score equal to the negative log of the *p*-value of the right-tailed Fisher’s exact test was assigned for each network. This score takes into account the number of eligible metabolites in our dataset and the size of the network to calculate the fit between each network and the metabolites in the dataset.

#### Cytoscape

Metabolomic networks were also generated using Cytoscape version 3.0.2 [75]. Cytoscape allows users to build and analyze networks of genes and compounds, identify enriched pathways from expression profiling data, and visualize changes in gene expression and/or compound concentration. Two plug-in apps were used for metabolic analyses directly from mass spectrometry data: (i) MetScape 3.0.0 [76] that traces connections between metabolites, reactions and genes, and provides a bioinformatics framework for the visualization and interpretation of metabolomics data; (ii) MetDisease 1.0.0 [77] that allows users to annotate a metabolic network with MeSH disease terms, explore related diseases within a network, and link to PubMed references corresponding to any node and selection network. MetScape uses an internal relational database stored at NCIBI to integrate metabolic compounds, reactions and pathway information from KEGG, EHMN, or Entrez Gene IDs. MetDisease supports both KEGG IDs and PubChem IDs. Metscape and MetDisease were used in addition to Ingenuity Pathway Analysis to interpret results of canonical pathways found by IPA.

#### Overlap/enrichment analyses

To assess the statistical significance of overlap/intersection of a set of metabolic compounds (e.g. a IPA functional/disease category) with another set of compounds (e.g. a IPA network), we tested the null hypothesis that the two sets of compounds are unrelated i.e. that any intersection is due to chance alone (i.e. the result of a random selection process). Using the hypergeometric distribution as the null distribution, and letting *X* be the random variable of the observed number of metabolic compounds in common between the two sets, for a given number *y* of compounds in the first set, *L* compounds in the second set, and a total of *N* compounds/molecules from the knowledge database that are associated with the analysis (the size of the reference set or “universe”), the probability of observing *X* = *x* overlapping compounds under the null is given by:

$$P\left(X=x|y,N,L\right)=\frac{\left(\begin{array}{c} y\\ x \end{array}\right)\left(\begin{array}{c} N-y\\ L-x \end{array}\right)}{\left(\begin{array}{c} N\\ L \end{array}\right)}$$

This rejection probability gives the *p*-value of overlap/intersection, based on the assumed null probability distribution.

## Results

### Grouping of plasma samples

**Figure 1:**
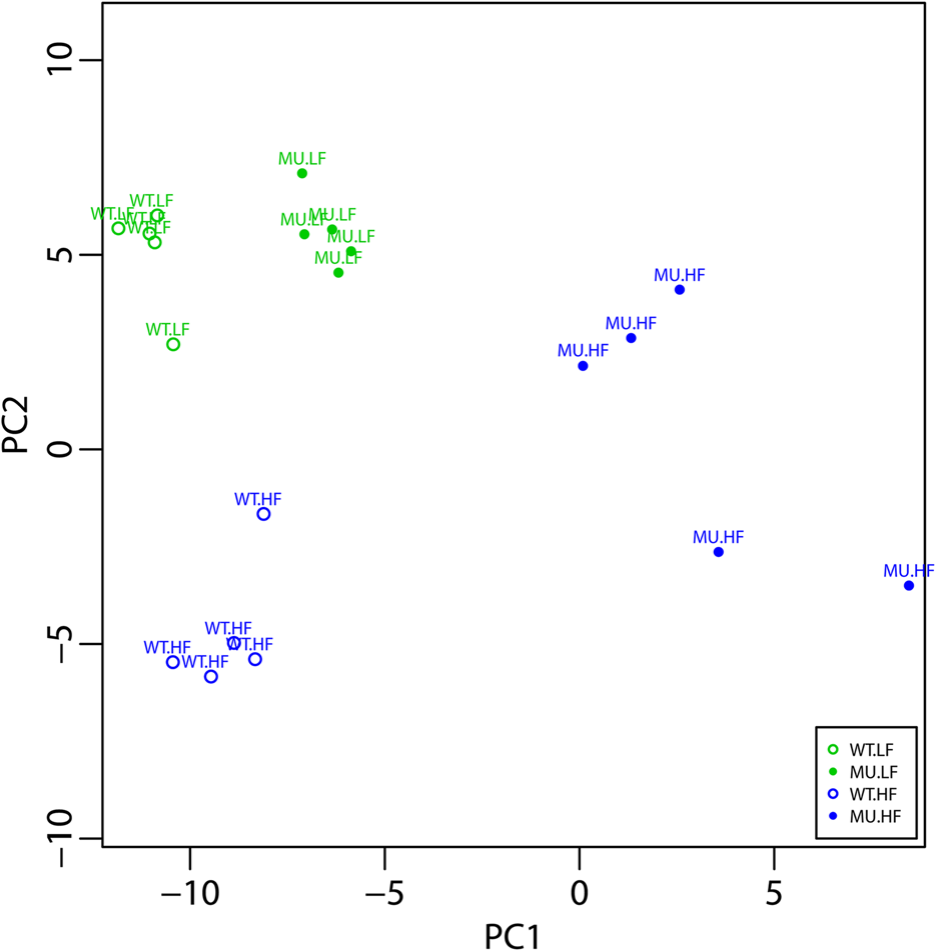
**Groups and outliers detection by 2D PCA scatterplot in plasma samples.** The 2D scatterplot uses the first two PCs to display the relationship between plasma samples (dots) as indicated by inter-individual distances. See ‘Methods’ section for the interpretation of samples and between-samples positions. Briefly, points that cluster together correspond to samples behaving similarly. Notice how samples form groups by experimental conditions and the absence of outliers. WT-LF, WT-HF, MU-LF, and MU-HF stand for the following groups: *Apc* Wild-Type - Low Fat Diet, *Apc* Wild-Type - High Fat Diet, *Apc* Mutant - Low Fat Diet, *Apc* Mutant - High Fat Diet respectively.

To study global metabolic differences associated with *Apc*^*Min/+*^ mutation and/or different diets we first used an unsupervised statistical approach. Principal Component Analysis (PCA) is a visualization and dimension reduction technique that allows detection of any groups or outliers in the data. The PCA was carried out in the plasma samples. Principal Components (PCs) derived from the analysis represent rotated coordinate axes pointing to directions of the predictor space (metabolite space) where the spread of the data (variance) is the largest, allowing a better visualization of groups or outliers in the samples (Figure 1). PCA scree plots determine the order and the number of principal components (PCs) accounting for the largest amount of variance in the data (Additional file 4: Figure S1). Here, a minimum of 2 PC’s was enough to explain most of the cumulative Percentage of Explained Variance for the plasma samples (PEV = 38.3%, PEV PC1 = 21.54%, PEV PC2 = 16.78%). The corresponding loading plots (Additional file 4: Figure S1) ranks the respective contribution of all the variables (metabolites) to each PC.

Based on the previous analysis of explained variance, the first two PCs were retained for groups and outliers detection among the samples. The biplot in Figure 1 is the PCA analysis of the plasma samples. It shows a complete separation between all four experimental groups (WT-LF, WT-HF, MU-LF, MU-HF). Notice, however, how the distance between Mutant vs. Wild-Type sample groups increases from Low-Fat diet to High Fat diet treatments. Similarly, notice how the distance between High Fat diet vs. Low-Fat diet sample groups increases from Wild-Type to Mutant treatments. Overall, this indicates a potential synergistic interaction effect between the *Diet* and *Genotype* factors in plasma metabolite concentration profiles, i.e. that a high fat diet tends to enhance metabolic differences associated with *Apc*^*Min/+*^ mutation, or vice-versa, i.e. that a *Apc*^*Min/+*^ mutation tends to enhance metabolic differences associated with a high fat diet. Essentially, this means that a single metabolic process may be affected by a combined treatment of a specific diet and genotype.

### Evaluation of treatments on metabolite concentration profiles in plasma

To profile the plasma metabolite concentrations across the experimental groups and determine their differential concentrations between experimental groups, we fitted the same linear mixed-effect model of analysis of variance univariately to each individual metabolite as described in the ‘Methods’ section. In this experimental design, the primary contrasts of interest are the *Genotype* and *Diet* main effects, as well as their *Genotype* by *Diet* interaction effect. The latter, although harder to interpret, is actually of most interest since this evaluates how a change in *Genotype* (e.g. from *Apc* Wild-Type to Mutant) affects a metabolite concentration and how this varies by type of *Diet* (High Fat vs. Low Fat); or alternatively, how a change of *Diet* (e.g. from Low Fat to High Fat) affects a metabolite concentration and how this varies by *Genotype* (*Apc* Wild-Type vs. Mutant). Metabolites were ranked by significance across comparisons. Adjusted *p*-values for multiple testing (or *q*-values) and a positive *pFDR* threshold cutoff of up to 5% were used to determine significance according to our criteria for confirmation of metabolite identification as described in the ‘Methods’ section (see also FDR analysis plots in Additional file 5: Figure S2). Note that, with the sizes of the lists (effects) of significant tests given below, a *pFDR* threshold cutoff of 5% means that no more than 4 to 5 metabolites, depending on the corresponding effect, are expected to be falsely called significant. The False Discovery Rate (*FDR*) theory, however, does not allow us to determine which metabolites are falsely included in each of these lists, but only their proportions [67].

Overall, a significant number of plasma metabolite concentrations changed by effect. These represent metabolites for which the concentration is sensitive to *Apc*^*Min/+*^ mutation, fat diet treatment or an interaction between the two. Venn diagrams summarize the counts of significant up- and down-regulated plasma metabolites. In the two main effects of interest, 97 plasma metabolites (61 up and 36 down) were found regulated between the *Genotype* groups; and 82 plasma metabolites (44 up and 38 down) between the *Diet* groups (Additional file 2: Table S2; Additional file 3: Table S3 and Additional file 6: Figure S3).

Further, to describe how the *Genotype* effect (*Apc* Wild-Type vs. Mutant) on a plasma metabolite concentration varies by the *Diet* (High Fat vs. Low Fat), or vice-versa, we focused on the *Genotype* by *Diet* interaction effect. In this interaction effect, 65 plasma metabolites (46 up and 19 down) were found differentially regulated. This *Genotype* by *Diet* interaction effect result is consistent with observations made for their individual main effects as the majority of metabolites identified as significant in the interaction effect were also found synergistically and/or antagonistically changed for these two factors individually. This is also consistent with the PCA interpretation (Figure 1). This result is summarized in a Venn diagram (Additional file 6: Figure S3) and the list is provided in Additional file 7: Table S4.

To visually describe the metabolites with significant regulation, it is convenient to visualize them in a so-called volcano plot. (Additional file 8: Figure S4). Overall, the lists of plasma regulated metabolites reveals the metabolomics variations resulting from the individual or combined effects of a mutation in the *Apc* gene and/or a high-fat diet. In the next section, we further examined how these metabolomics variations correlate to a clinical outcome of interest, namely the intestinal polyp counts.

### Independence between genotype and diet factors

Table 2 shows total polyp counts as measured in the intestine at animal sacrifice. On the one hand, wild-type animals do not apparently develop any polyps at all with either diet. This represents a classical situation of artificial over-inflation of zeros in count data analysis in the presence of relatively low counts and sample sizes. On the other hand, *all Apc*^*Min/+*^ mice show polyps growth, and high fat diet promotes polyps development (Table 2).

**Table 2:** Total polyp counts in the small intestine and colon by experimental groups

		**Diet factor (DF)**		
		**Low fat**	**High fat**	**Total**
Genotype Factor (GF)	Wild-Type	0	0	0
	Mutant	51	273	324
% (Mutant/Total)		100	100	100

We tested the association/dependence of the two main factors (*Diet* by *Genotype*) and their contribution to polyp counts (Table 2). The Null hypothesis that is to be tested for comparing two categorical factors in a 2-by-2 contingency table is:

$$H_{0}:\;\mathrm{The}\;\mathrm{Genotype}\;\mathrm{factor}\;\mathrm{is}\;\mathrm{independent}\;\mathrm{of}\;\mathrm{the}\;\mathrm{Diet}\;\mathrm{factor}.$$

The *χ*^2^-test with one degree of freedom (*df* = 1) yielded $\chi_{1}^{2}\gg 1$ with a *p*-value < 2.2 E-16. Therefore, we would reject the null hypothesis of independence, i.e. there is strong evidence of association of polyp counts with specific groups of *Diet* by *Genotype* factors.

### Relationship between polyp counts and metabolite profiles in plasma

A mere comparison of plasma metabolite levels between experimental groups cannot answer the causality question as to whether the observed differences arise from or contribute to polyposis and tumorigenesis. Typically, this question can only be addressed with respect to the controlled experimental variables of our design, namely the *Genotype* and *Diet* factors. If, however, one models the relationship between the polyp counts simultaneously with the plasma metabolite levels and the controlled experimental variables, one can analyze how changes in the *Genotype* and *Diet* factor levels modify this relationship and determine what the metabolite associated with these changes are.

For each metabolite, we fit a zero-inflated Generalized Linear Model (GLM) of polyp counts with respect to the categorical variables of the design matrix (*Genotype* and *Diet*) and each univariate continuous measurement of metabolite concentration (see ‘Methods’ section). With the restriction that this linear model will, by definition, only explore linear relationships, this allows modeling the (linear) correlation between polyp counts and each metabolite concentration profile for each combination of *Genotype* and *Diet* factor levels. Specifically, the primary combination of interest in this experimental design is the two-way interaction between *Genotype* and *Diet* since this represents how polyp count varies by metabolite concentration and how this relationship is influenced by the *Apc* genotype (Wild-Type vs. Mutant) and diet content (High vs. Low Fat). Individual *p-*values of the coefficient of interest were reported for each individual model (i.e. metabolite). Adjusted *p*-values represent a positive FDR (see *pFDR* definition in the ‘Methods’ section). With a maximum FDR of 5%, we found as many as 102 plasma metabolites having a significant correlation with polyp count in association with an interaction effect (Table 3). Many of the plasma metabolites listed are still uncharacterized or un-annotated (see complete list by tissue in Additional file 9: Table S5). This list reflects how deeply the combination of *Apc*^*Min/+*^ mutation and high fat diets is associated with the plasma metabolome and translates into intestinal polyps formation. They are essential to further determine the underlying metabolomics pathways of the host and the role of environmental factors in relation to the progression of the disease. These results are further analyzed and discussed.

**Table 3:** **List of annotated plasma metabolites having a significant correlation with polyp counts in association with a** ***Genotype*** **by** ***Diet*** **interaction effect**

**Metabolite**	**HMDB**	**CAS**	**KEGG**	**p.val**	**Adj.p.val**
Uracil	HMDB00300	66-22-8	C00106	2.555E-11	2.130E-10
Palmetoleic acid	HMDB03229	373-49-9	C08362	2.311E-02	3.533E-02
Serine	HMDB00187	56-45-1	C00065	5.215E-02	3.533E-02
Glycine 2TMS	HMDB00123	56-40-6	C00037	9.164E-02	3.533E-02
Glucose	HMDB00122	50-99-7	C00031	1.203E-01	3.533E-02
Methionine	HMDB00696	63-68-3	C00073	1.339E-01	3.533E-02
Palmitate	HMDB00220	57-10-3	C00249	1.419E-01	3.533E-02
Gluconic acid	HMDB00625	526-95-4	C00257	1.432E-01	3.533E-02
Stearic acid	HMDB00827	57-11-4	C01530	1.457E-01	3.533E-02
Tryptophane	HMDB00929	73-22-3	C02983	1.481E-01	3.533E-02
Glycerol-1-phosphate	HMDB00126	57-03-4	C00093	1.507E-01	3.533E-02
Erytrithol		149-32-6		1.595E-01	3.533E-02
Threonine	HMDB00167	72-19-5	C00188	1.735E-01	3.533E-02
Fumarate	HMDB00134	110-17-8	C00122	1.744E-01	3.533E-02
Hippuric acid	HMDB00714	495-69-2	C01586	1.786E-01	3.533E-02
1-Monopalmitin				1.829E-01	3.533E-02
Cholesterol	HMDB00067	57-88-5	C00249	1.924E-01	3.533E-02
Phenylalanine	HMDB00159	63-91-2	C00079	2.023E-01	3.533E-02
Glutamic acid	HMDB00134	110-17-8	C00122	2.046E-01	3.533E-02
Lysine	HMDB00182	56-87-1	C00047	2.158E-01	3.533E-02
Proline	HMDB00162	147-85-3	C00148	2.290E-01	3.533E-02
Creatinine	HMDB00562	60-27-5	C00791	2.331E-01	3.533E-02
Succinic acid	HMDB00254	110-15-6	C00042	2.355E-01	3.533E-02
2-Monopalmitin		19670-51-0		2.428E-01	3.533E-02
1,2 Dipalmitin		761-35-3		2.600E-01	3.533E-02
Norleucine	HMDB01645	327-57-1	C01933	2.633E-01	3.533E-02
Ascorbic acid	HMDB00044	50-81-7	C00072	2.716E-01	3.533E-02
Urea	HMDB00294	57-13-6	C00086	2.756E-01	3.533E-02
Nicotinamide	HMDB01406	98-92-0	C00153	2.932E-01	3.584E-02
Pyrophosphate	HMDB00250	14000-31-8	C00013	2.992E-01	3.584E-02
Alanine	HMDB00161	56-41-7	C00041	3.127E-01	3.670E-02
Ribose-5-phosphate	HMDB01548	3615-55-2	C00117	3.287E-01	3.756E-02
1,3-Dipalmitin	HMDB31011	502-52-3		3.372E-01	3.763E-02
2-Amino adipic acid	HMDB00510	542-32-5	C00956	3.440E-01	3.763E-02
Pyroglutamic acid	HMDB00267	98-79-3	C01879	3.553E-01	3.763E-02
Glutamine	HMDB00641	56-85-9	C00064	3.568E-01	3.763E-02
Pyruvate	HMDB00243	127-17-3	C00022	3.569E-01	3.763E-02
Valine	HMDB00883	72-18-4	C00183	3.790E-01	3.769E-02
Inositol phosphate	HMDB34220	551-72-4	C06151	3.879E-01	3.769E-02
Malic acid	HMDB00744	6915-15-7	C00711	4.790E-01	4.158E-02
Glycerol	HMDB00131	56-81-5	C00116	5.514E-01	4.642E-02
Glycine	HMDB00123	56-40-6	C00037	5.824E-01	4.805E-02

Raw *p*-values from the Generalized Linear Model are reported with *pFDR*-adjusted *p*-values as described in the ‘Methods’ section. Metabolites with *pFDR* ≤ 0.05 are shown and ranked by *pFDR*-adjusted or equivalently by raw *p*-values. Accession numbers from the Human Metabolome Database accession (HMDB), the Kyoto Encyclopedia of Genes and Genomes (KEGG) databases, and Chemical Abstract Service (CAS) are provided. Only annotated compounds of the list of significant compounds are shown here. The full list with hyperlinked accession numbers is provided in (Additional file 9: Table S5).

**Figure 2:**
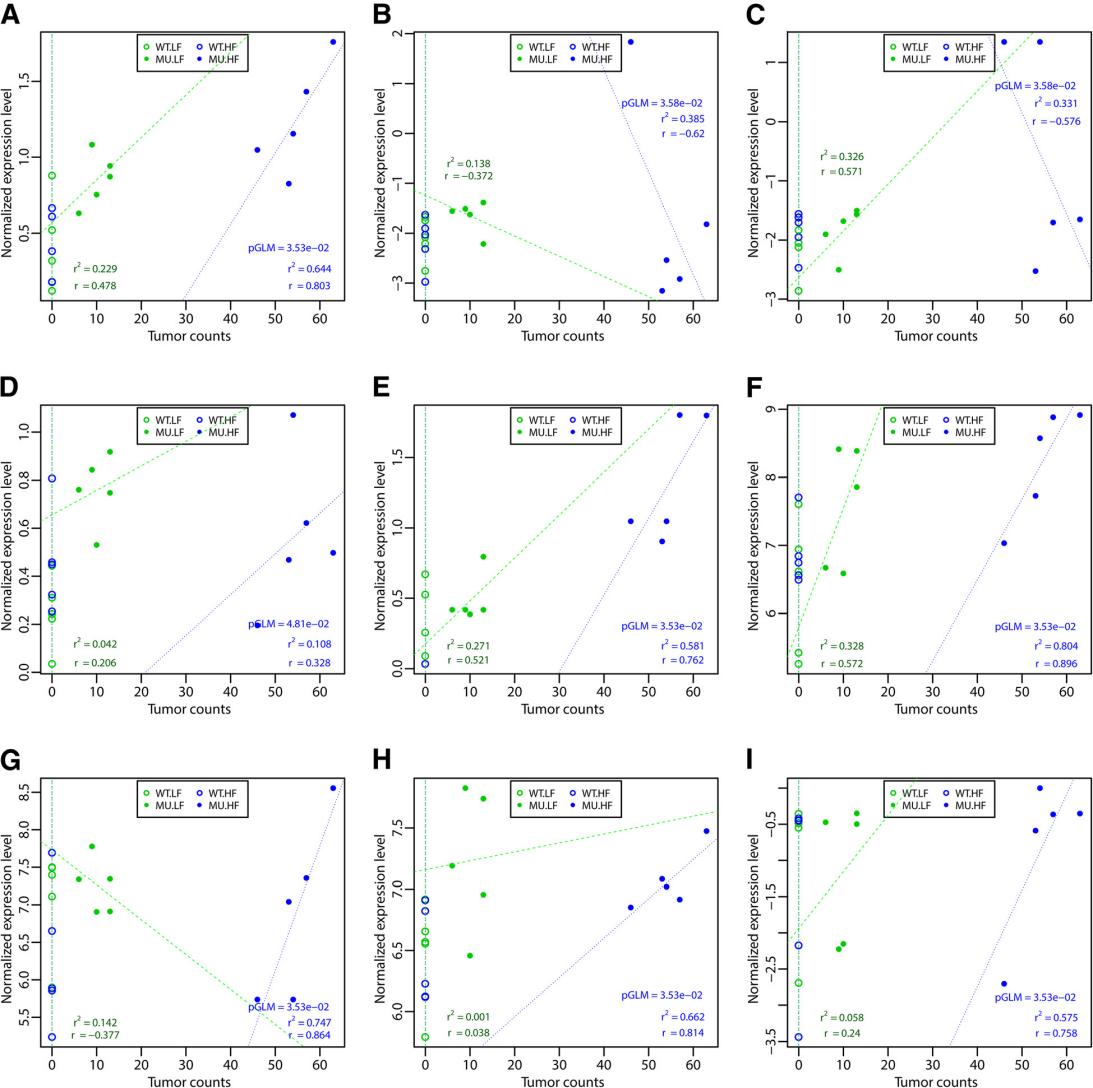
**Selected plasma metabolites having a significant correlation with polyp counts in association with the*Genotype by Diet*interaction effect.** The nine metabolites were selected from Table 3 of significant plasma metabolites having a correlation with polyp counts in association with a *Genotype* by *Diet* interaction effect. They reflects how the combination of ApcMin/+ mutation and high fat diets is associated with the plasma metabolome and translates into intestinal polyps formation. In all subplots **A**-**I**, the correlation p-values (pGLM) were obtained from fitting the GLM after adjustment for multiplicity (‘see ‘Methods’ and Table 3). To graphically show the grouping and localization of the samples as well as to visualize the linearity and correlation at play for each significant metabolite, the linear regression line is plotted (dotted lines) with its corresponding determination coefficient (r2) and the Pearson correlation coefficient (ρ). Because of the vertical alignment of all sample points from the WT-LF and MU-LF groups, the resulting coefficient of determination (r2) is 0 and the Pearson correlation coefficient (ρ) is mathematically undetermined. In contrast, for all the other samples in the MU-LF and MU-HF experimental groups, where r2 and ρ are both meaningful, we observe for all metabolites **(A-I)** the best coefficient of determination (r2) and the largest Pearson correlation coefficient (ρ) in the MU-HF group (blue) in comparison to the MU-LF group (green). Hippuric acid **(A)**, Pyrophosphate **(B)**, Nicotinamide **(C)**, Glycine **(D)**, Phenylalanine **(E)**, Methionine **(F)**, Tryptophane **(G)**, Threonine **(H)**, Glutamic acid **(I)** for all combinations of Genotype and Diet factors. WT-LF, WT-HF, MU-LF, and MU-HF stand respectively for the following groups: Apc Wild-Type - Low Fat Diet, Apc Wild-Type - High Fat Diet, Apc Mutant - Low Fat Diet, Apc Mutant - High Fat Diet. Concentration levels are normalized on a transformed scale as explained in the ‘Methods’ section.

As an illustration of the above association and influence of controlled experimental variables, we show correlation and regression results between plasma metabolite levels and polyp counts by combination of *Genotype* and *Diet* factor levels for the hippuric acid, the pyrophosphate and nicotinamide metabolic pathways (Figure 2A,B,C) and the uptake of six amino acids (Figure 2D,E,F,G,H,I). Consistent with previous results, note immediately how the Mutant - High Fat Diet samples specifically cluster in the region of the plot of higher polyp counts (Figure 2). Further, note also for the same experimental group of samples the positive (increase) or negative (decrease) correlation of polyp counts with plasma metabolite levels. The plot also shows the comparisons of correlation coefficients and determination coefficients observed in each of the combination of *Genotype* and *Diet* factors levels (Figure 2). These results are further analyzed and discussed.

### Functional analyses in plasma

Plasma metabolites correlated with intestinal polyps and associated with a *Genotype* by *Diet* interaction effect (Table 3 and complete Additional file 9: Table S5) were subjected to functional metabolomics annotations analyses. For reasons explained above, this list of identified metabolites is essential to the understanding of the underlying metabolomics pathways related to the progression of intestinal tumorigenesis and how genetic and environment factors affect it.

Biological functions, canonical metabolomics pathways and metabolomics interaction networks analyses were first carried out by Ingenuity Pathway Analyses (IPA). Remarkably, results show that among the complete list of significant canonical biological functions/diseases found by IPA, the top two ones are annotated as (in order of significance): “*Cancer*” and “*Gastrointestinal Disease*” (complete list in Additional file 10: Table S6(A)). Also, among the lists of significant IPA canonical metabolomics pathways, the “*tRNA Charging*” pathway is the most significant (complete list in Additional file 10: Table S6(B)).

**Figure 3:**
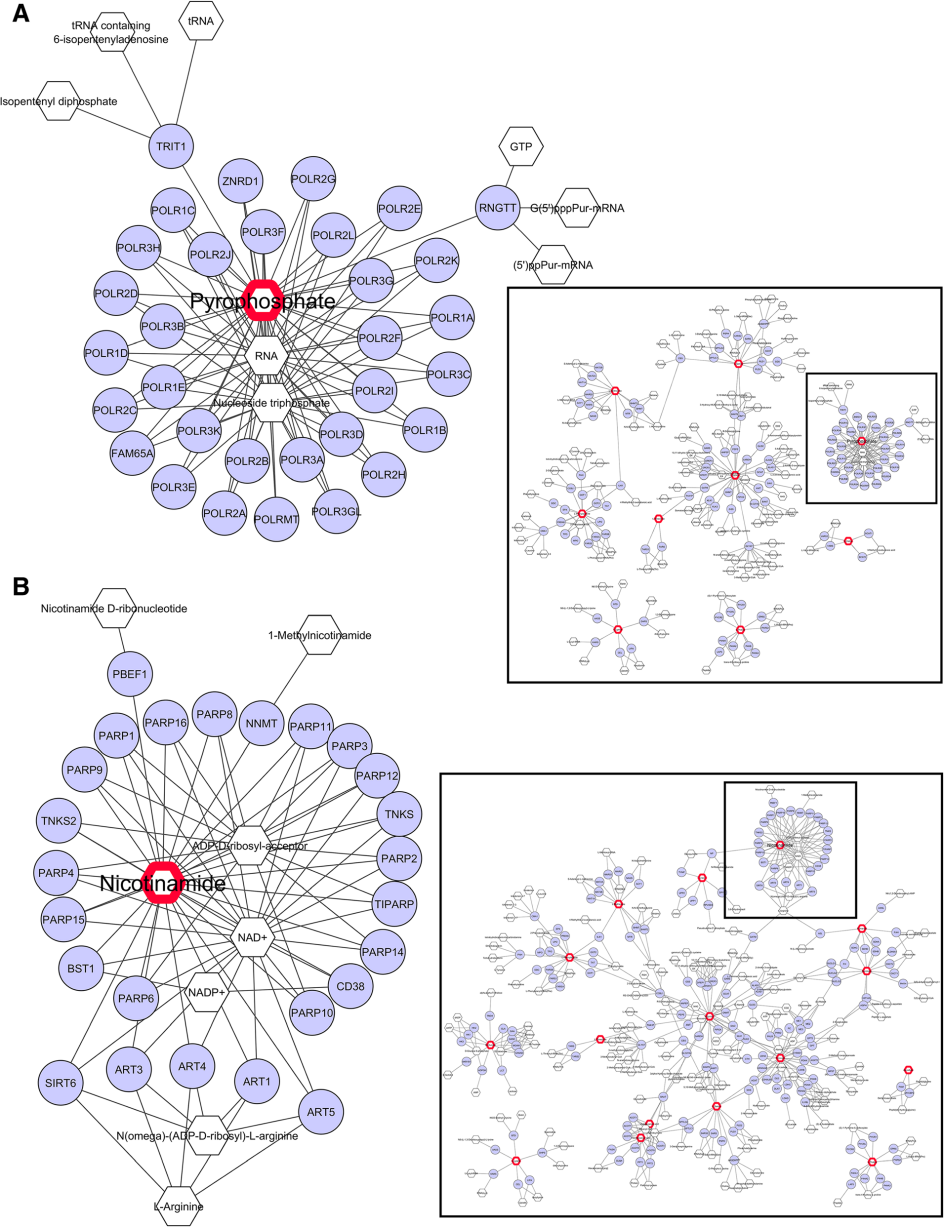
**Metscape gene-compounds metabolic network views of top IPA canonical pathway and biological function/disease.** Integrated Metscape genes-compounds metabolic network view for **(A)** the “*tRNA Charging*” canonical pathway and **(B)** the “*Gastrointestinal Cancer*” canonical function/disease found by Ingenuity Pathway Analysis (Additional file 10: Table S6). Close-up views (top left and bottom left) are excerpts of the corresponding global Metscape genes-compounds network views (lower right). Hexagonal nodes (transparent) and circle nodes (blue) represent metabolic compounds and genes, respectively. Hexagonal nodes with red border paintings indicate metabolites present in our list (plasma metabolites listed in Table 3 and Additional file 9: Table S5). Notice the high node degree of these networks (number of connections a node has to other nodes) and the two most prominent hub configurations formed by Pyrophosphate (top left) in relation to numerous RNA polymerase genes **(A)** and by Nicotinamide (bottom left) in relation to numerous PARP genes **(B)**.

Integrating molecular networks from high-throughput data is often sought as a powerful means to visualize and model functional interactions in a system of molecular components. To further elucidate the biological meaning of the above “*tRNA Charging*” canonical pathway as well as the canonical function/disease “*Cancer and Gastrointestinal Disease*” found by IPA, Metscape genes-metabolites metabolic networks were built using their corresponding metabolic compounds. In each network view, connections between metabolites and genes were drawn to form a unified conceptual network as described in the Methods section. Genes-metabolites networks corresponding to the “*tRNA Charging*” canonical pathway and to the “*Cancer and Gastrointestinal Disease*” canonical disease revealed key ‘hub’ compounds also present in our list of interest (Table 3 and complete Additional file 9: Table S5); such as, respectively, pyrophosphate, in relation to numerous genes of the RNA polymerases family (Figure 3A), and nicotinamide, in relation to numerous genes of the poly(ADP-ribose) polymerases (PARP) families, as well as Sirt-6 histone deacetylase (Figure 3B). These findings are further discussed. An important property of networks or graphs is related to the node connectivity or node degree, i.e. the number of connections a node has to other nodes. In non-random networks the degree distribution or probability distribution of these degrees over the whole network follows a scale-free power law rather than a binomial distribution [78]. This so-called ‘scale-free’ connectivity property is conjectured to be present in most common networks such as biological, genetic, metabolic, social networks and the Internet. In any kind of network, the presence of hierarchical structures such as ‘hubs’ and the associated overall high level node connectivity (node degree) are hallmarks of non-randomness. Here, the figure shows this ‘hub’ feature in at least two prominent metabolite compounds of interest (pyrophosphate, Figure 3A, nicotinamide, Figure 3B) as well as the overall high level of node degree in both genes-metabolites networks.

**Figure 4:**
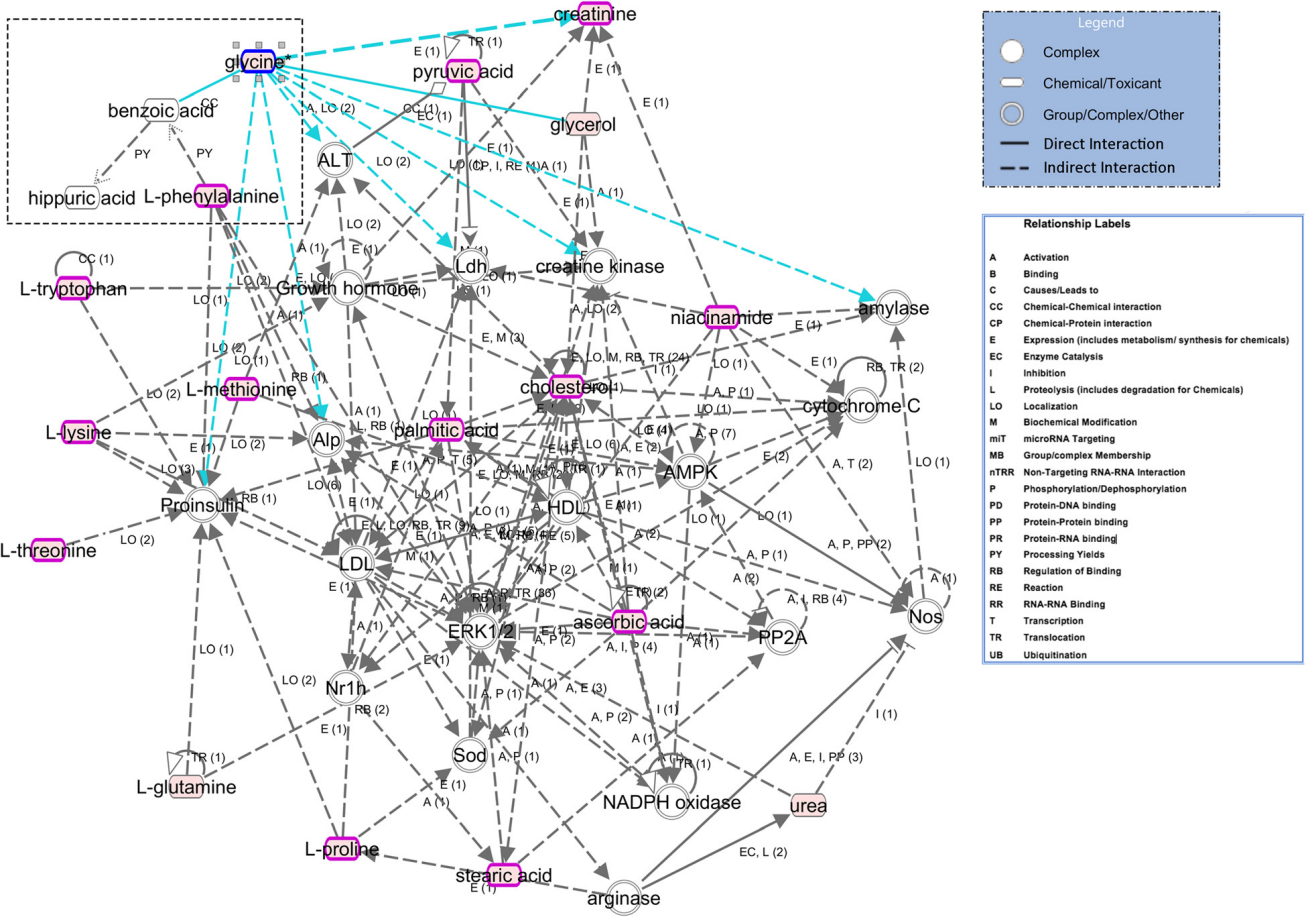
**IPA metabolic interaction network of plasma metabolites having a significant correlation with polyp counts in association with a*Genotype*by*Diet*interaction effect.** Most significant IPA metabolic interaction network for the plasma metabolites correlated with polyp counts and associated with a *Genotype* by *Diet* interaction (listed in Table 3 and Additional file 9: Table S5). For metabolic pathways, an arrow pointing from node A to node B signifies that B is produced from A. The keys of molecule shapes and relationship labels show the nature of the nodes as and their relationships, respectively. Acronyms refer to relationship labels and numbers in parentheses next to them refer to the number of literature findings that support these relationships individually. Both direct (solid line) and indirect (broken line) specific relationships between the metabolites are indicated. Nodes with pink fillings represent metabolites that were significantly changed in the interaction effect and correlated with the outcome of interest. Metabolites involved in “*Gastrointestinal Disease and Cancer*” are circled in dark pink. Note in the top-left of the graph the presence of the hippuric acid metabolic pathway resulting from the conjugation of glycine with benzoic acid, which in turn is a conversion by-product of the L-phenylalanine metabolism.

We further analyzed the function of plasma metabolites correlated with intestinal polyps and associated with a *Genotype* by *Diet* interaction effect (Table 3 and complete Additional file 9: Table S5) by building IPA interaction metabolomics networks. Among the list of significant interaction metabolomics networks found by IPA (complete list in Additional file 11: Table S7), the most significant one is canonically referred to as “*Increased Levels of Albumin, Cellular Growth and Proliferation, Organismal Development”* (Figure 4). To add functional description to this biological network, we overlaid the information of the top two biological functions/diseases found by IPA, namely “*Cancer”* and *“Gastrointestinal Disease*”. Notice the extent of overlap of this metabolomics interaction network with the disease set (13/37 nodes). The corresponding intersection *p*-value (*p* = 6.27E-06) was computed as described in the Methods section (here with *x* = 13 and parameters *y* = 18, *L* = 37 and *N* = 150), which is statistically highly significant at the *α* = 0.05 significance level, indicating that the two sets of compounds are (probably) truly intersecting. Therefore, in addition to showing the specific metabolites (and their relationships) that have a *Genotype* by *Diet* Interaction effect associated with polyp counts, this biological network shows the extent of overlap between these metabolites and the disease information, i.e. their relevance to the disease process. Furthermore, the biological network also shows the involvement of the hippuric acid metabolic pathway (Figure 4), and consequently its relevance to the “*Cancer and Gastrointestinal Disease*” canonical function/disease mentioned above. A biological model for this finding is proposed and further discussed.

Finally, to gain insight into the underlying metabolic and genetic networks involving the plasma metabolites correlated with intestinal polyps and associated with a *Genotype* by *Diet* interaction effect (Table 3 and Additional file 9: Table S5), we subjected these metabolites to integrated Metscape-MetDisease disease annotation analyses as described in the Methods section. MeSH terms corresponding to “*Gastrointestinal Neoplasms”* and “*Gastrointestinal Disease”* were matched to a metabolites-only network (Additional file 12: Figure S5A) or a genes-metabolites network (Additional file 12: Figure S5B). The overlap between the set of metabolic compounds and their annotation to the disease terms is striking. In the former case, a total of 45 matching nodes were found out of a total of 249 metabolic compounds nodes (Additional file 12: Figure S5A). The corresponding overlapping *p*-value was computed here with parameters *x* = 45, *y* = 124 (total number of unique nodes, matched to all MeSH terms for the network), *L* = 249 (metabolic compounds nodes for the network) and *N* = 2136 (total number of compounds in Metscape for which there is pathway information). The resulting *p*-value (0) that is below the computer lower bound of floating-point representation let us draw two conclusions. First, the metabolomics signature of “*Gastrointestinal Neoplasms”* and “*Gastrointestinal Disease”* is certainly present in the list of metabolic compounds of interest (Table 3 and Additional file 9: Table S5). Second, and more importantly, the metabolic network views provided in (Additional file 12: Figure S5B) are useful representations of the metabolite-metabolite interactions sub-networks and of the metabolite-genes workflows that underlie the progression of intestinal polyposis or tumorigenesis and how genetic and environment factors affect it. Further, the figure shows hierarchical structures such as ‘hubs’ for many metabolite compounds of interest. This feature and the overall high level of node degree in both networks (Additional file 12: Figure S5A and B) are hallmarks of organized non-random networks.

### Acyl-CoA profiles in liver

**Figure 5:**
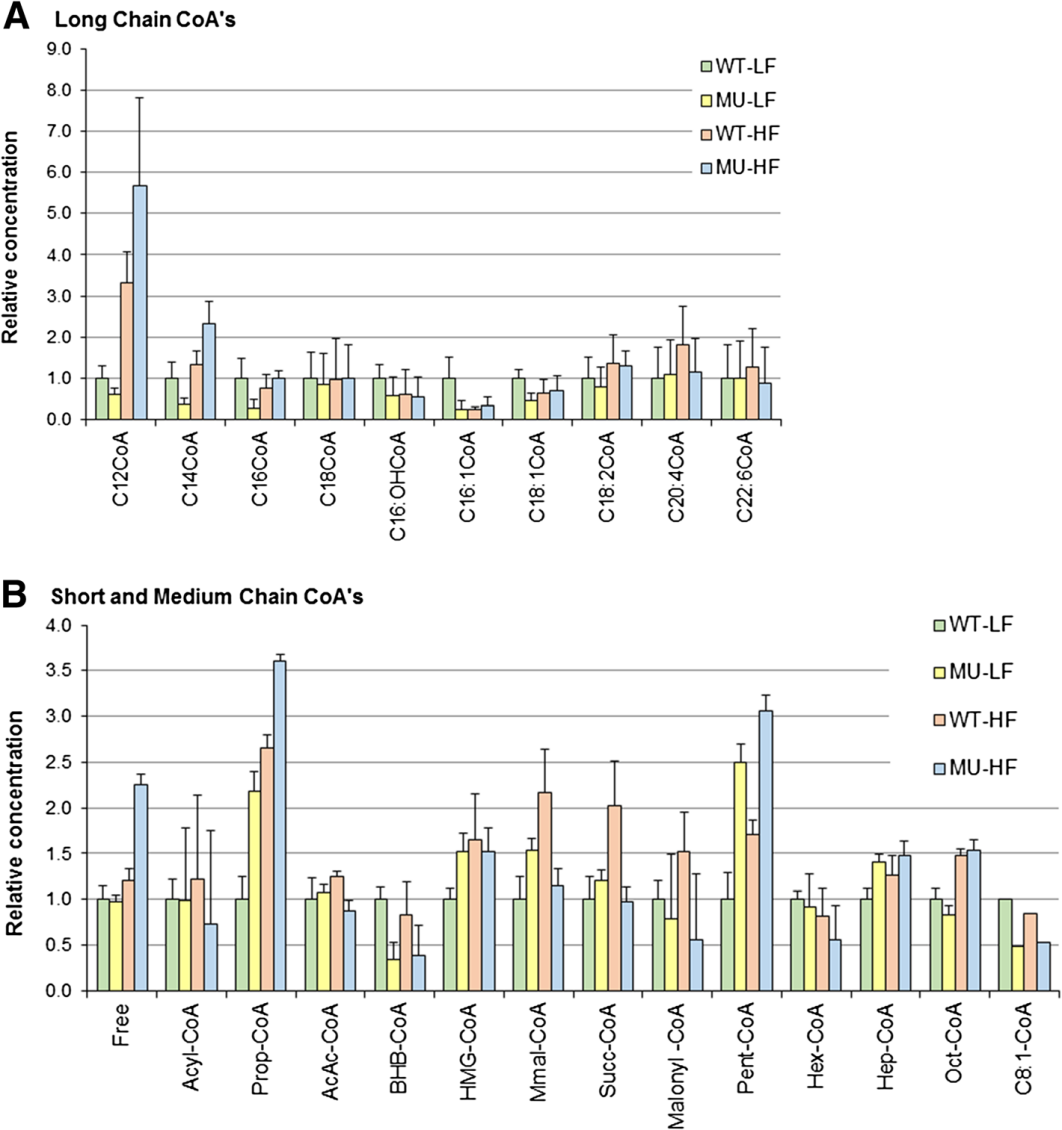
**Bar chart of mean concentrations of Acyl-CoAs by group in liver. (A)** Mean concentrations of long-chain and **(B)** short/medium-chain acyl-CoAs were calculated per experimental group (*n*_*g*_ = 5) and normalized per gram of wet liver tissue [nmol/gr] and per mean concentration in the WT-LF group for all combinations of *Genotype* and *Diet* factors. All WT-LF CoA’s relative mean concentrations are therefore equal to 1. Standard error of the means are shown with the ANOVA *p*-value for assessing the significance of difference of group means as compared to the overall mean. In the legend WT-LF, WT-HF, MU-LF, and MU-HF stand respectively for the following groups: *Apc* Wild-Type - Low Fat Diet, *Apc* Wild-Type - High Fat Diet, *Apc* Mutant - Low Fat Diet, *Apc* Mutant - High Fat Diet.

**Figure 6:**
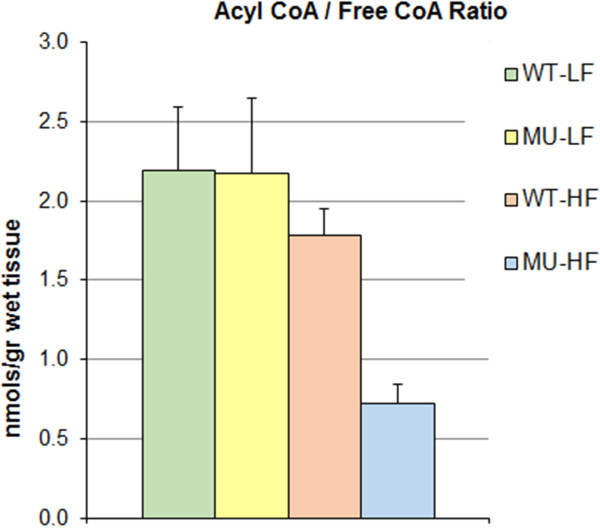
**Bar chart of mean concentration ratios of [Acyl-CoA]/[CoASH] by group in liver.** Mean concentration ratios were calculated per experimental group (*n*_*g*_ = 5) and normalized per gram of wet liver tissue [nmol/gr]. Standard errors of the means are shown with the ANOVA *p*-value for assessing the significance of difference of group means as compared to the overall mean. In the legend, WT-LF, WT-HF, MU-LF, and MU-HF stand respectively for the following groups: *Apc* Wild-Type - Low Fat Diet, *Apc* Wild-Type - High Fat Diet, *Apc* Mutant - Low Fat Diet, *Apc* Mutant - High Fat Diet.

Cancer progression is usually associated with profound changes in energy metabolism since tumor cells stimulate growth through increased glycolysis and fatty acid oxidation pathways [79]. Because acyl-CoAs represent important intermediates of lipid metabolism that are affected in cancer [29], we report the concentrations of both long-chain as well as short/medium-chain acyl-CoAs from the liver tissue. Mean absolute concentrations (measured by LC-MS/MS) were normalized per gram of wet liver tissue [nmol/mg] (Table 4, Figures 5 and 6). Relative concentrations were calculated with respect to acyl-CoAs concentrations in *Apc* Wild-Type Low Fat (WT-LF) animals (Figure 5).

**Table 4:** Variations of mean total long chain Acyl-CoAs concentrations by group in liver

**Genotype – diet combination**	**WT - LF**	**MU - LF**	**WT - HF**	**MU - HF**
	1.16 ± 0.23	0.41 ± 0.07	1.27 ± 0.23	1.52 ± 0.25

Mean concentrations were calculated per experimental group (*n*_*g*_ = 5), normalized per gram of wet liver tissue [nmol/mg] and computed for the following long-chain acyl-CoAs: C_12_-CoA, C_14_-CoA, C_16_-CoA, C_18_-CoA, C_16:OH_-CoA, C_16:1_-CoA, C_18:1_-CoA, C_18:2_-CoA, C_20:4_-CoA, C_22:6_-CoA. Standard error of the means are indicated. WT-LF, WT-HF, MU-LF, and MU-HF stand respectively for the following combination of treatments: *Apc* Wild-Type - Low Fat Diet, *Apc* Wild-Type - High Fat Diet, *Apc* Mutant - Low Fat Diet, *Apc* Mutant - High Fat Diet.

#### Long-chain Acyl-CoAs

Total content of long chain acyl-CoAs data show that the effect of high fat feeding is associated with a very significant fold change between MU-HF and MU-LF groups (*FC* = 3.66; *p* = 6.52 E-6), while no significant difference is observed between WT-HF and WT-LF (*FC* = 1.09; *p* = 0.714) (Table 4). Overall, this profile underscores the synergistic (interaction) effect of the *Apc*^*Min/+*^ mutation with a high fat diet in the total content of long chain acyl-CoAs.

First, looking further into individual long chain acyl-CoAs, note that the effect of high fat feeding is associated with an increase of specific long chain acyl-CoAs contents, namely C_12_-CoAs and to a lower extent C_14_-CoAs, in both *Apc* genotype backgrounds: We report in *Apc* wild-type and *Apc*^*Min/+*^ mutant animals, both fed with high fat, a significant fold change increase of C_12_-CoAs content as compared to all other long chain acyl-CoAs contents (WT-HF group: *FC* = 3.34; *p* = 3.06 E-4; MU-HF group: *FC* = 4.23; *p* = 1.30 E-4) (Figure 5A). Second, note the remarkable fold change increase of C_12_-CoAs content upon high fat feeding in *Apc*^*Min/+*^ mutant animals (fold-change between MU-LF and MU-HF experimental groups: *FC* = 9.55; *p* = 3.68 E-5), which is less pronounced in *Apc* Wild-Type animals (fold-change between WT-LF and WT-HF experimental groups: *FC* =4.02; *p* = 1.13 E-3) (Figure 5A). The latter profile also underscores the synergistic (interaction) effect of the *Apc*^*Min/+*^ mutation with a high fat diet in the content of C_12_-CoAs.

#### Short and medium-chain Acyl-CoAs

We found a very significant fold change increase of free acyl-CoAs contents in the MU-HF group as compared to the other three experimental groups (*FC* = 2.1; *p* = 3.48 E-3), underlying a strong synergistic (interaction) effect of *Apc*^*Min/+*^ mutation with a high fat diet on these compound levels (Figure 5B). Likewise, note the synergistic (interaction) effects of *Apc*^*Min/+*^ mutation and high fat diet in the increase of propionyl-CoA (*FC* = 2.86; *p* = 6.78 E-2) and pentanoyl-CoA (*FC* = 2.47; *p* = 4.67 E-2) levels, indicating that the *Apc*^*Min/+*^ mutation and high fat diet act both alone and in concert on these compound levels (Figure 5B). Finally, note the increasing (main) effect of high fat diet on octanoyl-CoA levels (*FC* = 1.65; *p* = 8.37 E-4) and the decreasing (main) effect of *Apc*^*Min/+*^ mutation in BHB-CoA levels (*FC* = 1 / 2.53 ≈ 0.39; *p* = 7.39 E-4) (Figure 5B).

#### Acyl-CoA/Free-CoA ratios

The [Acyl-CoA]/[CoASH] ratio usually reflects energy demand of the tissue. This ratio alters in response to change in energy state of a system. Figure 6 shows the [Acyl-CoA]/[CoASH] ratios for all four groups. Effects of increased availability of free fatty acids on β-oxidation rates and on gene expression of β-oxidation enzymes have already been studied by others [80,81]. These studies illustrate that high levels of dietary fatty acids induce mitochondrial and peroxisomal β-oxidation in liver and thus decrease the [Acyl-CoA]/[CoASH] ratio. Our findings are compatible with those studies and show a significant decrease in the [Acyl-CoA]/[CoASH] ratio (*FC* = 1/2.87 ≈ 0.35; *p* = 2.42 E-3) for the MU-HF group (Figure 6). This indicates a synergistic effect of the *Apc*^*Min/+*^ mutation with a high fat diet on the relative abundance of Acyl-CoAs to free-CoAs.

## Discussion

Our study demonstrates that global GC-MS-based plasma metabolomics and targeted LC-MS/MS-based liver metabolite profiling can be combined with clinical observations to investigate *diet interventions* and *genetic susceptibility* to intestinal cancer. Our results underscore the high potential of metabolomics profiling in pattern recognition and characterization of potential pathways of intestinal cancer. Metabolites from different pathways have been identified including TCA cycle intermediates, amino acids, carbohydrates, lipids and various acyl-CoAs. Unsupervised and supervised statistical procedures allowed us to study plasma metabolic alterations between wild type and genetically predisposed mice to intestinal cancer (*Apc*^*Min/+*^) under diet intervention. As a part of our study, we were able to correlate an important clinical outcome of intestinal cancer to plasma metabolic profiles in an animal model genetically predisposed to intestinal cancer, and determine how this is modified by a change of diet. In our experiment, this correlation characterizes how polyp counts in the small intestine vary by metabolite concentration, levels of the *Genotype* factor (*Apc* Wild-Type vs. Mutant), and levels of the *Diet* factor (High Fat vs. Low Fat). Overall, plasma metabolomics concentration profile results indicate that that high-fat diet significantly enhances some of the metabolic perturbations that are associated with *Apc*^*Min/+*^ mutation and small intestine tumor development.

Increase in some of plasma amino acids levels has been observed. Since cancer cells are high proliferation cells and require free amino acids as an energy source [82] or as building blocks for metabolites [83], presumably elevated plasma amino acids levels reflect malignant tissues need for bloodstream amino acid supplies. Regarding the role of methionine, there are controversial studies showing either a positive or negative effect of methionine on different types of cancer in various tissues [84-87]. The methionine-mediated DNA methylation along with folate and homocysteine (through S-adenosyl methionine) hypomethylation could increase the risk of cancer [88].

While poly(ADP-ribose) polymerase-1 (PARP-1) has well-described functions in the regulation of chromatin structure, transcription and genomic integrity, recent evidences point to a role in transcriptional regulation in the context of human malignancy (reviewed in [89,90]). Specifically, PARP-1 may play an important role in carcinogenesis of colorectal cancer and recent findings have raised the possibility of using PARP inhibitor therapy in colorectal cancers clinical trials (reviewed in [91]). The observed negative correlation between nicotinamide concentration levels and polyp numbers (Table 3, Figure 2C), along with the numerous known relations of nicotinamide to genes of the PARP family that were revealed in our data (Figure 3A), suggest a possible antagonist effect of nicotinamide compound levels on polyp formation and on promotion of intestinal cancer via inhibition of PARP activity. Alternatively, the reduction of nicotinamide concentration could reflect its consumption as substrate for Nicotinamide phosphoribosyltransferase (NAmPRTase or Nampt) to synthesize nicotinamide phosphoribosyl pyrophosphate as a key precursor for synthesis of NAD^+^[92]. NAD^+^ is required to support tumor growth, both as substrate for PARP [91] and for function of SIRT1, an NAD^+^ dependent deacetylase whose activity is associated with deacetylation of p53, consequently leading to progressive tumor growth [93]. The potential consumption of nicotinamide to support NAD^+^ synthesis and increased activity of PARP and/or SIRT1 indicates this pathway, which was identified in our studies, is a potentially important target for chemotherapy.

**Figure 7:**
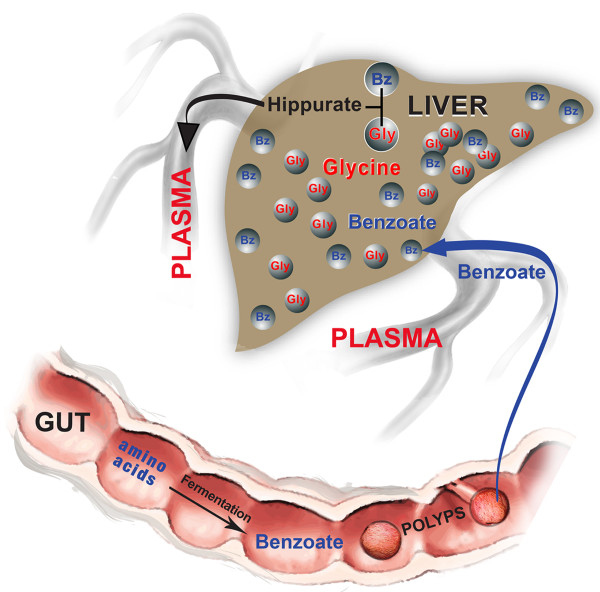
**Proposed biological model involving hippuric acid metabolism.** This model derives from the most significant IPA metabolic interaction network plotted in Figure 4 in relation to the plasma metabolites correlated with polyp counts and associated with a *Genotype* by *Diet* interaction (listed in Table 3 and Additional file 9: Table S5). The sketch displays the workflow of benzoic acid (benzoate - Bz) uptake by intestinal polyps, followed by its transport to the liver, its reaction with glycine (Gly) to produce hippuric acid (hippurate) and its final release in the plasma.

The correlation between plasma hippuric acid (hippurate or benzoyl-glycine) levels and polyp numbers could be a result of different rates of benzoate uptake, possibly through polyps. Berger *et al.* reported in their study that benzoic acid is absorbed from the intestine by sodium-coupled monocarboxylate transporters (SMCTs), followed by benzoyl-glycine production in liver from benzoic acid through activation of benzoate to benzoyl CoA [94]. SLC5A8 and SLC5A12 transporters mediate uptake of a variety of monocarboxylates including benzoic acid and show different concentration profiles in normal *vs.* cancer tissues [95]. Consequently, we interpret the increase in plasma hippurate concentration as reflecting a stimulation of benzoate uptake from the intestine, probably linked to the monocarboxylate transporter associated with intestinal polyps (See our putative biological model in Figure 7).

A key question relates to the causality of our findings with intestine polyp formation. Should some of the plasma metabolite levels found in animal fed with high fat diet be considered, at least in part, a cause or a consequence of the formation of these polyps? Further studies are warranted to determine whether the increase of plasma metabolites levels that is observed in the combination of high-fat diet with *Apc* mutation and for which there is a significant correlation with intestinal polyp formation (Table 3 and complete Additional file 9: Table S5), could be interpreted as a consequence of tumor formation and not a cause. In some cases, such as hippurate metabolism, this interpretation would be consistent with our proposed biological model for that compound (Figure 7) and support our recent results in the same *Apc*^*Min/+*^ mouse model of intestinal neoplasia in that high-saturated fat-diets increase polyp development and formation [48]. Alternatively, a possibility that needs to be considered stems from recent studies showing that intestinal and circulating metabolites may be significantly altered by the intestinal microbiome to change the composition and available energy content of ingested nutrients as well as to generate factors which stimulate inflammation, cardiovascular disease and cancer [96-98].

To investigate deregulations in lipid metabolism, which are critical for energy homeostasis, we have measured liver acyl-CoA profiles. High fat diet intervention stimulates fatty acid metabolism by increasing the availability of free fatty acids that might lead to the increase in β-oxidation rates. On the other hand, apart from the nutrition intervention, the fatty acid oxidation pathway is up-regulated in cancer cells since these cells have high proliferation rates and increased energy consumption. Our findings show a decrease of [acetyl-CoA]/[CoASH] ratio and it presumably reflects up-regulation in β-oxidation pathway as a result of a combination of *Apc*^*Min/+*^-mediated cancer progression and high fat diet.

## Conclusions

Our study shows that mass spectrometry-based cancer metabolomics, when used in an appropriate experimental design, can give important insights into genotype characterization and diet intervention effects, and their association with intestinal polyposis and tumorigenesis. Although high-throughput mass spectrometry-based metabolomics data feature *relative* concentrations (peak area of analyte/peak area of references compound), these studies show that high-throughput metabolomics combined with appropriate statistical modeling and large scale functional approaches can be used to monitor and infer changes and interactions in the metabolome and genome of the host under controlled experimental conditions. Further these studies demonstrate the impact of diet on metabolic pathways and its relation to intestinal cancer progression. Based on our results, metabolic signatures of polyposis intestinal carcinoma have been identified, such as those involving nicotinamide and hippuric acid metabolic pathways, which may serve as a useful targets for the development of therapeutic interventions.

### Supporting information

The online version of this article contains five (5) additional figures and seven (7) additional tables for a total of 12 Additional files.

## Abbreviations

PCA: Principal Component Analysis; PC: Principal component; PEV: Percentage of Explained Variance; ANOVA: Analysis of variance; GLM: Generalized Linear Model; GC-MS: Gas chromatography–mass spectrometry; LC-MS: Liquid chromatography-mass spectrometry; APC: Adenomatous polyposis coli; WT: Wild-type; MU: Mutant; DF: Diet factor; GF: Genotype factor; TF: Source of tissue factor; HF: High fat; LF: Low fat; PLA: Plasma; LIV: Liver.

## Competing interests

The authors declare they have no competing interests.

## Authors’ contributions

J-ED designed and performed all the statistical and functional analyses. YS performed all the biochemical and mass spectrometric assays. SD conducted all the animal and genetic work. All authors (J-ED, YS, SD, NAB, HB) formulated the problem, wrote and approved the manuscript.

## Supplementary Material

Additional file 1: Table S1 Specific Multiple Reaction Monitoring (MRM) Transitions for Each Acyl-CoA. Free-CoA and all CoA esters show an m/z transition of 507 amu during LC-MS/MS analysis. Click here for file

Additional file 2: Table S2 Full List of Significant Plasma Metabolites in the *Genotype* Effect. Significant metabolites are ranked by significance and highlighted in yellow (controlled at *pFDR* ≤ 5%). Statistics that are listed are described in the ‘Methods’ section: estimated log_2_-Fold Change (logFC), moderated *t*-, and *B*- statistics, raw and pFDR-adjusted p-values. Metabolites are ranked by adjusted *p*-value and then by *B*-statistic. Click here for file

Additional file 3: Table S3 Full List of Significant Plasma Metabolites in the *Diet* effect. Significant metabolites are ranked by significance and highlighted in yellow (controlled at *pFDR* ≤ 5%). Statistics that are listed are described in the ‘Methods’ section: estimated log_2_-Fold Change (logFC), moderated *t*-, and *B*- statistics, raw and *pFDR*-adjusted *p*-values. Metabolites are ranked by adjusted *p*-value and then by *B*-statistic. Click here for file

Additional file 4: Figure S1 Scree Plots and Loading Plots for the Plasma Samples. (A) Plot of the distribution of contributed variances (i.e. eigenvalues based on the spectral decomposition of the correlation matrix) by Principal Component PC# 1 – 20. (B) Cumulative Percent of Explain Variance (PEV) against the number of selected Principal Components 1–20 PC’s. Dashed red lines on both plots show the corresponding contributed variance (33.8) and cumulative PEV (38.3%) for the first two selected PC’s. Loading plots of the top 100 metabolites loadings ordered by decreasing absolute correlation coefficient with the corresponding selected Principal Component (PC1 (C), and PC2 (D)). Click here for file

Additional file 5: Figure S2 FDR Analysis Results by Effect for the Plasma Samples. *Genotype Effect* (GF) or *Diet Effect* (DF) and their *Interaction Effect* (GFDF) are plotted for the Plasma samples. (A) Positive *pFDR*-controlled discoveries by effect, where *pFDR* is controlled under some dependency at 5%. (B) Expected number of false discovery by effect under a *pFDR* of 5%. (C) Comparison of raw *p*-values vs. adjusted *p*-values (*q*-values) by effect. Click here for file

Additional file 6: Figure S3 One-set and Three-set Venn Diagrams by Effect for the Plasma Samples. Each Venn diagram shows the distribution of counts (pFDR ≤ 5%) of plasma metabolites regulated by effect (circle or set). Counts are given for the three classical effects of interest: (A, E) main Genotype effect; (B, F) main Diet effect; (C, G) Genotype by Diet interaction effect; (D, H) their three-set intersections. The counts in each Venn diagram of the bottom row (E, F, G, H) represent the number of regulated metabolites by effect and by direction of change, either up (red) or down (green). One may obtain the aggregated counts of up- and down-regulated metabolites in each of the one-set Venn diagram (A, B, C, D) by summing the up and down counts in the corresponding Venn diagram below, provided that this is done by effect and not by intersection subset alone (duplicates are accounted for by effect when intersections are formed between multiple effects in multiple-set Venn diagrams). For instance, the aggregated count of up- and down-regulated metabolites in the Diet effect is 82, that is, in the one-set Venn diagrams (B, F): 82 (B) = 42 + 38 (F), which also matches the counts in the three-set Venn diagrams (D, H): 82 = 14 + 12 + 51 + 5 (D) = (14 + 23) + (11 + 9) + (12 + 5) + (7 + 1) (H). The total number of regulated metabolites in all effects is given by the aggregated counts in the top row Venn diagrams (A, B, C, D), that is, 33 + 12 + 14 + 1 + 51 + 5 + 8 = 124. MU-WT, HF-LF, and MU-WT x HF-LF stand respectively for the following groups: Apc Mutant vs. Wild Type, High vs. Low Fat Diet, and Apc Mutant vs. Wild Type in High vs. Low Fat Diet. Click here for file

Additional file 7: Table S4 Full List of Significant Plasma Metabolites in the *Genotype* by *Diet* Interaction Effect. Significant metabolites are highlighted in yellow (controlled at *pFDR* 5%). Statistics that are listed are described in the ‘Methods’ section: estimated log_2_-Fold Change (logFC), moderated *t*-, and *B*- statistics, raw and *pFDR*-adjusted *p*-values. Metabolites are ranked by adjusted *p*-value and then by *B*-statistic. Click here for file

Additional file 8: Figure S4 Volcano Plots of Significant Plasma Metabolites by Effect in Plasma. The *Genotype* (A) and *Diet* (B) main effects are shown with the *Genotype* by *Diet* interaction effect (C) in plasma samples. When only two group samples are compared at a time, a volcano plot is adequate. The volcano plot is a scatter plot of all metabolite species arranged by an individual measure of magnitude of change of concentration between experimental groups (horizontal axis) versus a corresponding measure of statistical significance (vertical axis). Here, the horizontal axis represents the estimated log-Fold-Change of differential expression, denoted log_2_(*FC*) or *M*. The vertical axis represents the log-Odds of differential concentration, denoted log_2_(*Odds*) or *B*. Each point on the volcano plot represents a metabolite. Metabolites with large absolute values of estimated Log_2_-Fold Changes (*logFC* or *M*) *and* large values of Log_2_-odds (*B*) indicate metabolites with significant differential concentrations in the contrast or effect of interest*.* All preselected metabolites (201) are plotted in grey, but only those with a significant effect (controlled at *pFDR* ≤ 5%) are highlighted in red (up-regulated) or green (down-regulated). Points on the volcano plot in the upper right and upper left directions are metabolites with large absolute values of estimated Log_2_-Fold Changes on the transformed scale (log_2_(*FC*) or *M*) *and* large values of Log_2_-odds (log_2_(*Odds*) or *B*), indicating significantly regulated metabolites. Click here for file

Additional file 9: Table S5 Full List of Plasma Metabolites Having a Significant Correlation with Polyp Counts in association with a *Genotype* by *Diet* Interaction Effect. Raw and pFDR-adjusted p-values are reported as described in the ‘Methods’ section. Metabolites are ranked by pFDR-adjusted p-values or equivalently by raw p-values, both from the Generalized Linear Model. Accession numbers from the Human Metabolome Database accession (HMDB), the Kyoto Encyclopedia of Genes and Genomes (KEGG) databases, and Chemical Abstract Service (CAS) are provided. Click here for file

Additional file 10: Table S6 Full List of Significantly Enriched IPA Metabolic Biological Functions/Diseases and Canonical Pathways. Both sub-tables are from the results of significant plasma metabolites correlated with polyp counts and associated with the *Genotype* by *Diet* interaction effect (Table 3 and Additional file 9: Table S5). (A) List of top 12 significant IPA canonical biological functions. (B) List of top 10 significant IPA canonical metabolic pathways. ‘BH *p*-value’ stands for enrichment *p*-value, adjusted by the FDR control procedure (see ‘Methods’ section), and ‘95% CI’ is its corresponding 95% confidence interval. ‘Ratio’ in a given pathway represents the overlap of those metabolites found in our Table 3 to all those constitutive of the corresponding canonical pathway. ‘Molecules’ represents the ones found in our Table 3 matching up the corresponding canonical pathway. For both sub-tables, ranking was done by BH-adjusted enrichment *p*-values. The FDR threshold of significance was set at 5%. Click here for file

Additional file 11: Table S7 Full List of Significant IPA Metabolic Interaction Networks. List of top 3 significant metabolic interaction networks from the results of significant plasma metabolites correlated with polyp counts and associated with the *Genotype* by *Diet* interaction effect (Table 3 and Additional file 9: Table S5). Click here for file

Additional file 12: Figure S5 Full-Size High-Resolution Graphs of the Cytoscape Compounds-only and Genes-Compounds Metabolic Networks. Integrated Metscape-MetDisease metabolic network views of (A) metabolic compound-only and (B) genes-compounds for the plasma metabolites correlated with polyp counts and associated with a Genotype by Diet interaction (listed in Table 3 and Additional file 9: Table S5). Hexagonal nodes (transparent) and circle nodes (blue) represent metabolic compounds and genes, respectively. Hexagonal nodes with red border paintings indicate metabolites present in our list (plasma metabolites listed in Table 3 and Additional file 9: Table S5). Hexagonal nodes with yellow fillings represent metabolite compounds whose MeSH disease annotation matches the terms “*Gastrointestinal Disease*” or “*Gastrointestinal Neoplasm*”. Notice the high node degree of each of these networks (number of connections a node has to other nodes) and the extent of overlap of MeSH-disease annotated-metabolite compounds in both networks. Click here for file
