# Supplementary figures and images for: Metabolomics of Apc Min/+ mice genetically susceptible to intestinal cancer
Source: BMC Syst Biol. 2014 Jun 23;8:72. doi: 10.1186/1752-0509-8-72 (PMC4099115; doi:10.1186/1752-0509-8-72)

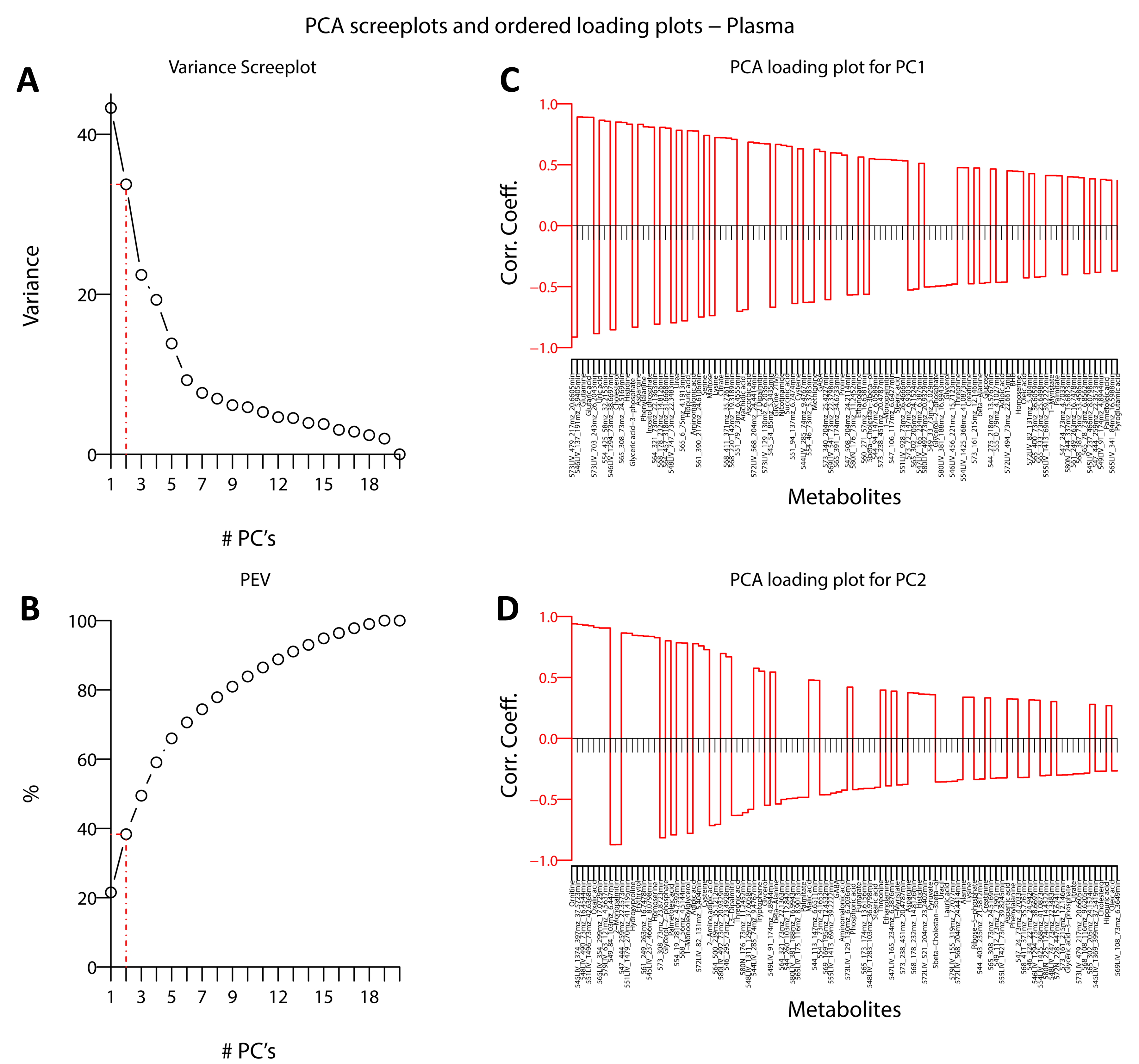

Supplement: Additional file 4: Figure S1 — Scree Plots and Loading Plots for the Plasma Samples. (A) Plot of the distribution of contributed variances (i.e. eigenvalues based on the spectral decomposition of the correlation matrix) by Principal Component PC# 1 – 20. (B) Cumulative Percent of Explain Variance (PEV) against the number of selected Principal Components 1–20 PC’s. Dashed red lines on both plots show the corresponding contributed variance (33.8) and cumulative PEV (38.3%) for the first two selected PC’s. Loading plots of the top 100 metabolites loadings ordered by decreasing absolute correlation coefficient with the corresponding selected Principal Component (PC1 (C), and PC2 (D)). [file 1752-0509-8-72-S4.tiff]

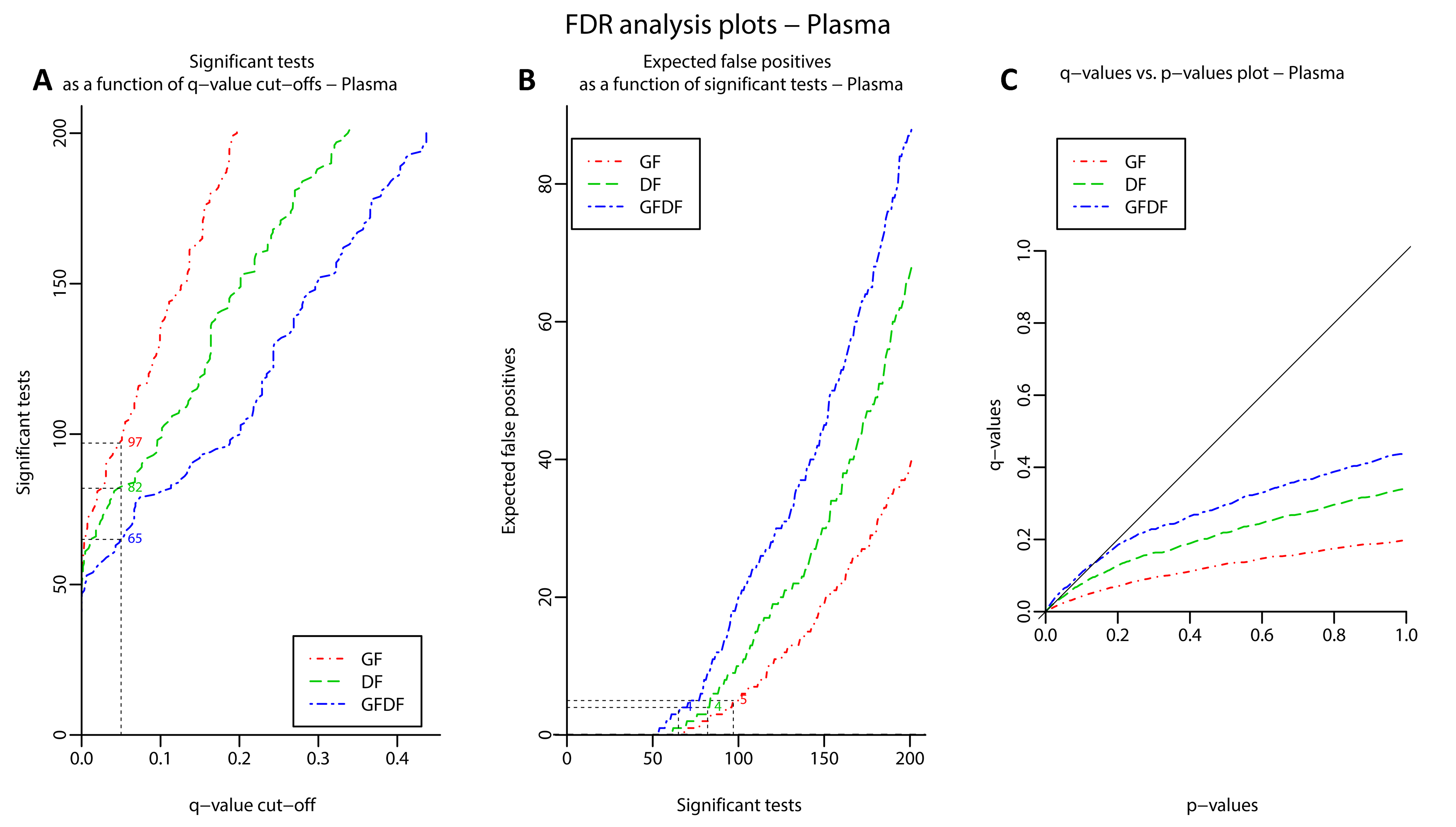

Supplement: Additional file 5: Figure S2 — FDR Analysis Results by Effect for the Plasma Samples. Genotype Effect (GF) or Diet Effect (DF) and their Interaction Effect (GFDF) are plotted for the Plasma samples. (A) Positive pFDR-controlled discoveries by effect, where pFDR is controlled under some dependency at 5%. (B) Expected number of false discovery by effect under a pFDR of 5%. (C) Comparison of raw p-values vs. adjusted p-values (q-values) by effect. [file 1752-0509-8-72-S5.tiff]

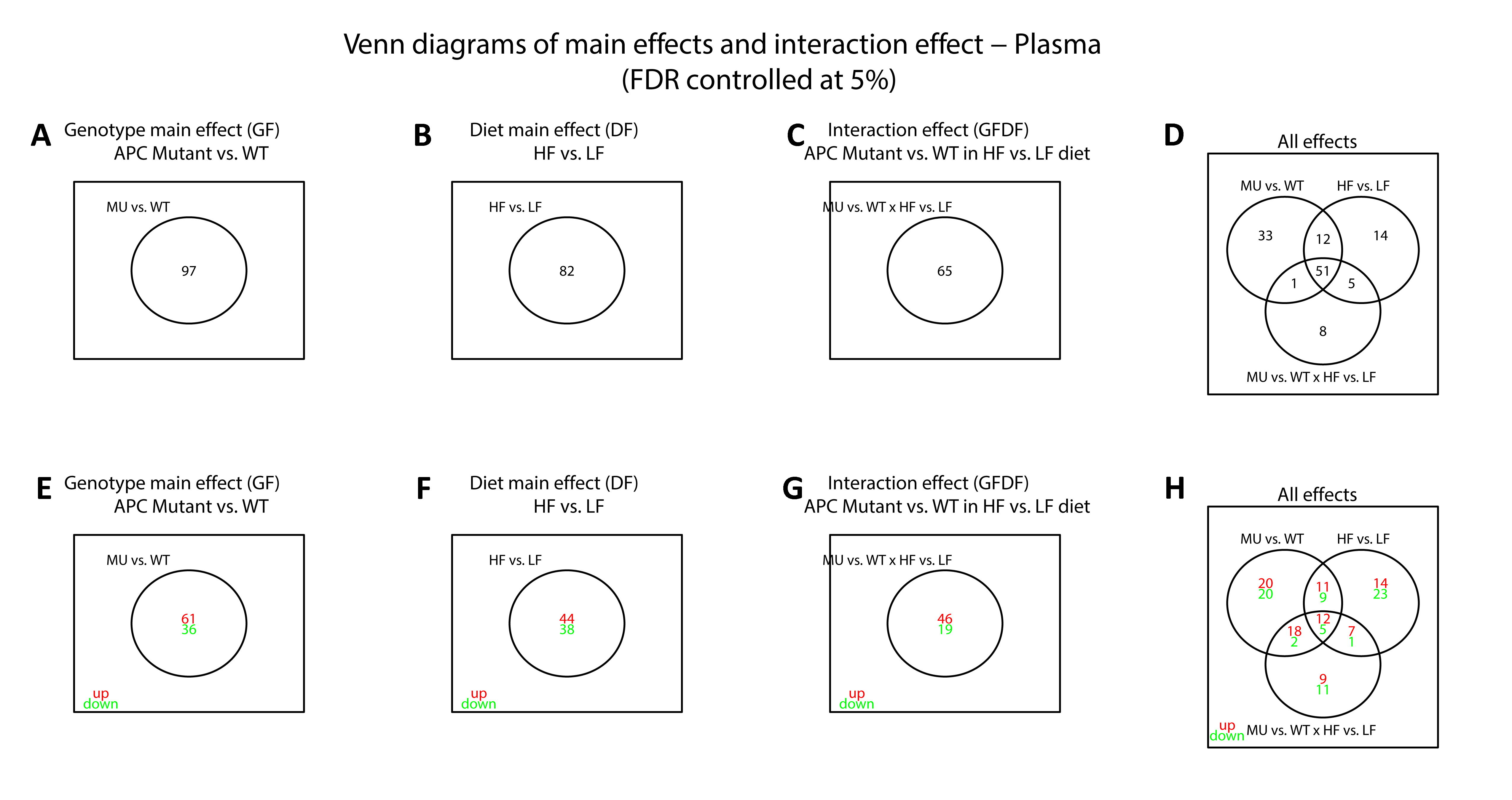

Supplement: Additional file 6: Figure S3 — One-set and Three-set Venn Diagrams by Effect for the Plasma Samples. Each Venn diagram shows the distribution of counts (pFDR ≤ 5%) of plasma metabolites regulated by effect (circle or set). Counts are given for the three classical effects of interest: (A, E) main Genotype effect; (B, F) main Diet effect; (C, G) Genotype by Diet interaction effect; (D, H) their three-set intersections. The counts in each Venn diagram of the bottom row (E, F, G, H) represent the number of regulated metabolites by effect and by direction of change, either up (red) or down (green). One may obtain the aggregated counts of up- and down-regulated metabolites in each of the one-set Venn diagram (A, B, C, D) by summing the up and down counts in the corresponding Venn diagram below, provided that this is done by effect and not by intersection subset alone (duplicates are accounted for by effect when intersections are formed between multiple effects in multiple-set Venn diagrams). For instance, the aggregated count of up- and down-regulated metabolites in the Diet effect is 82, that is, in the one-set Venn diagrams (B, F): 82 (B) = 42 + 38 (F), which also matches the counts in the three-set Venn diagrams (D, H): 82 = 14 + 12 + 51 + 5 (D) = (14 + 23) + (11 + 9) + (12 + 5) + (7 + 1) (H). The total number of regulated metabolites in all effects is given by the aggregated counts in the top row Venn diagrams (A, B, C, D), that is, 33 + 12 + 14 + 1 + 51 + 5 + 8 = 124. MU-WT, HF-LF, and MU-WT x HF-LF stand respectively for the following groups: Apc Mutant vs. Wild Type, High vs. Low Fat Diet, and Apc Mutant vs. Wild Type in High vs. Low Fat Diet. [file 1752-0509-8-72-S6.tiff]

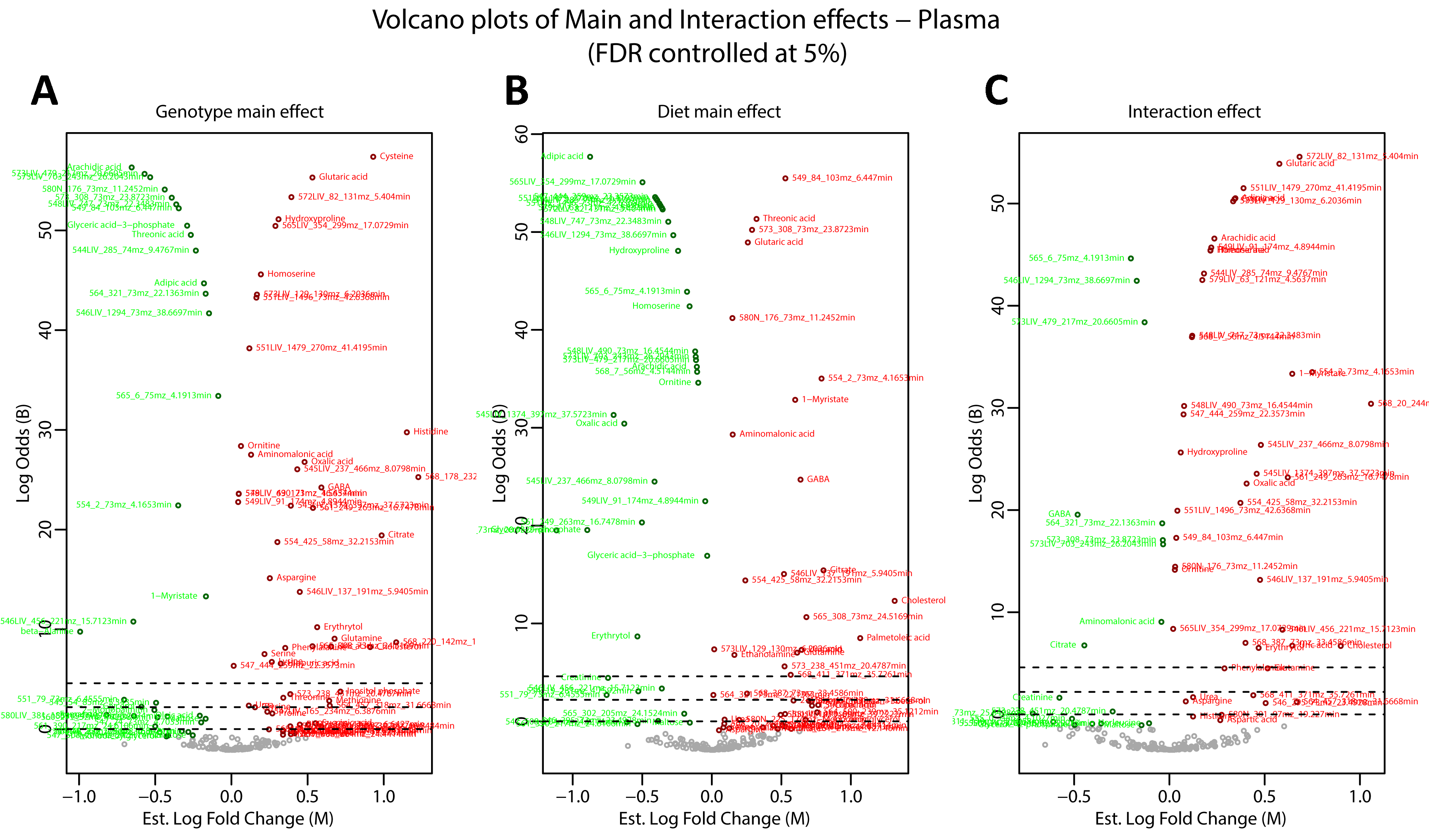

Supplement: Additional file 8: Figure S4 — Volcano Plots of Significant Plasma Metabolites by Effect in Plasma. The Genotype (A) and Diet (B) main effects are shown with the Genotype by Diet interaction effect (C) in plasma samples. When only two group samples are compared at a time, a volcano plot is adequate. The volcano plot is a scatter plot of all metabolite species arranged by an individual measure of magnitude of change of concentration between experimental groups (horizontal axis) versus a corresponding measure of statistical significance (vertical axis). Here, the horizontal axis represents the estimated log-Fold-Change of differential expression, denoted log2(FC) or M. The vertical axis represents the log-Odds of differential concentration, denoted log2(Odds) or B. Each point on the volcano plot represents a metabolite. Metabolites with large absolute values of estimated Log2-Fold Changes (logFC or M) and large values of Log2-odds (B) indicate metabolites with significant differential concentrations in the contrast or effect of interest. All preselected metabolites (201) are plotted in grey, but only those with a significant effect (controlled at pFDR ≤ 5%) are highlighted in red (up-regulated) or green (down-regulated). Points on the volcano plot in the upper right and upper left directions are metabolites with large absolute values of estimated Log2-Fold Changes on the transformed scale (log2(FC) or M) and large values of Log2-odds (log2(Odds) or B), indicating significantly regulated metabolites. [file 1752-0509-8-72-S8.tiff]

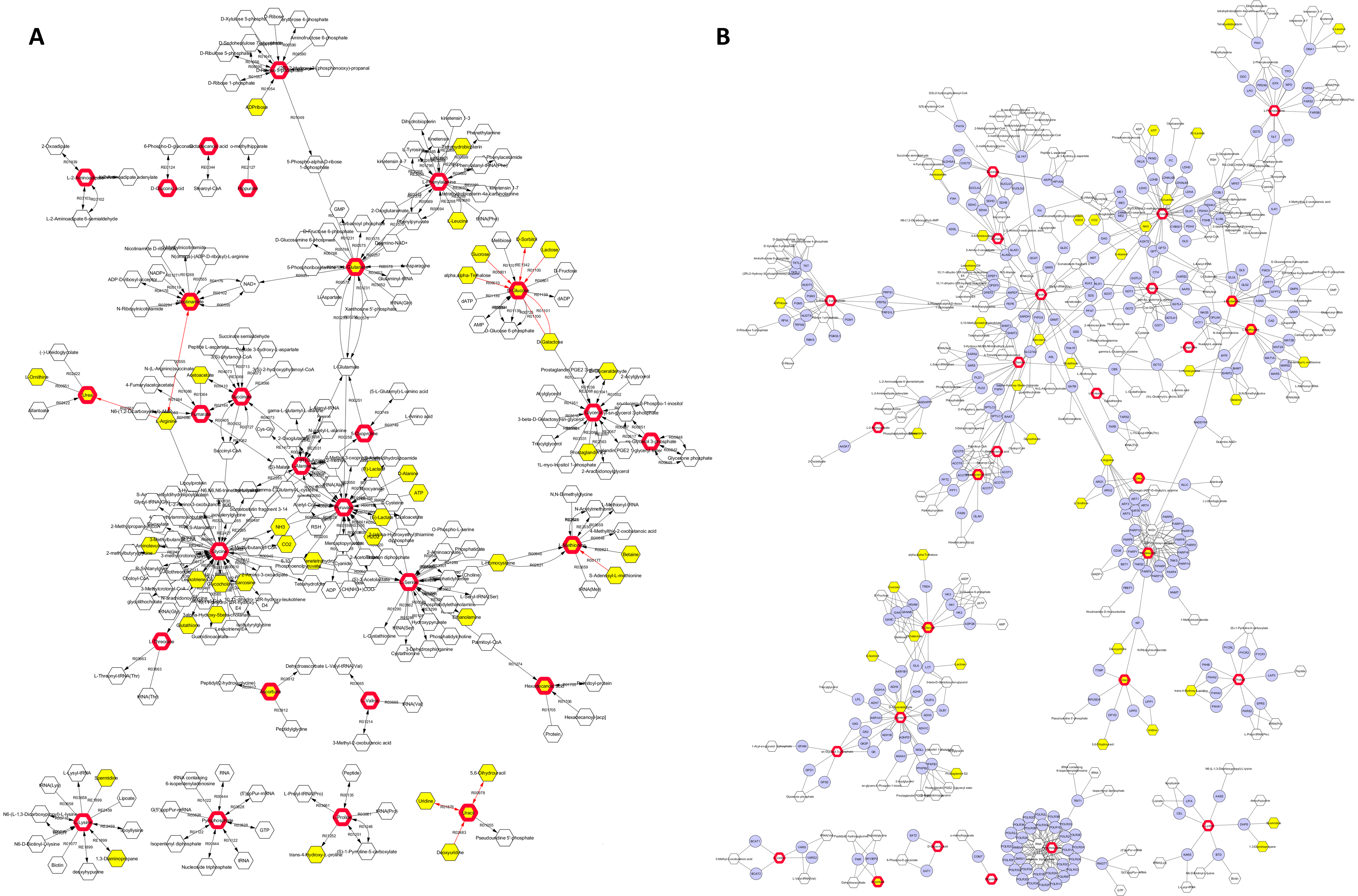

Supplement: Additional file 12: Figure S5 — Full-Size High-Resolution Graphs of the Cytoscape Compounds-only and Genes-Compounds Metabolic Networks. Integrated Metscape-MetDisease metabolic network views of (A) metabolic compound-only and (B) genes-compounds for the plasma metabolites correlated with polyp counts and associated with a Genotype by Diet interaction (listed in Table 3 and Additional file 9: Table S5). Hexagonal nodes (transparent) and circle nodes (blue) represent metabolic compounds and genes, respectively. Hexagonal nodes with red border paintings indicate metabolites present in our list (plasma metabolites listed in Table 3 and Additional file 9: Table S5). Hexagonal nodes with yellow fillings represent metabolite compounds whose MeSH disease annotation matches the terms “Gastrointestinal Disease” or “Gastrointestinal Neoplasm”. Notice the high node degree of each of these networks (number of connections a node has to other nodes) and the extent of overlap of MeSH-disease annotated-metabolite compounds in both networks. [file 1752-0509-8-72-S12.tiff]
